# Jean-Marie Souriau’s Symplectic Foliation Model of Sadi Carnot’s Thermodynamics

**DOI:** 10.3390/e27050509

**Published:** 2025-05-09

**Authors:** Frédéric Barbaresco

**Affiliations:** Thales, 91120 Palaiseau, France; frederic.barbaresco@thalesgroup.com

**Keywords:** thermodynamics, second principle, symplectic foliation, Riemannian foliation, Lie groups, Lie algebra cohomology, entropy, Casimir function, Pfaff forms

## Abstract

The explanation of thermodynamics through geometric models was initiated by seminal figures such as Carnot, Gibbs, Duhem, Reeb, and Carathéodory. Only recently, however, has the symplectic foliation model, introduced within the domain of geometric statistical mechanics, provided a geometric definition of entropy as an invariant Casimir function on symplectic leaves—specifically, the coadjoint orbits of the Lie group acting on the system, where these orbits are interpreted as level sets of entropy. We present a symplectic foliation interpretation of thermodynamics, based on Jean-Marie Souriau’s Lie group thermodynamics. This model offers a Lie algebra cohomological characterization of entropy, viewed as an invariant Casimir function in the coadjoint representation. The dual space of the Lie algebra is foliated into coadjoint orbits, which are identified with the level sets of entropy. Within the framework of thermodynamics, dynamics on symplectic leaves—described by the Poisson bracket—are associated with non-dissipative phenomena. Conversely, on the transversal Riemannian foliation (defined by the level sets of energy), the dynamics, characterized by the metric flow bracket, induce entropy production as transitions occur from one symplectic leaf to another.

## 1. Preamble

In this article, we develop the conceptual framework of foliations associated with the Pfaff equations of thermodynamics, in order to interpret the second principle of Sadi Carnot in light of recent developments in the symplectic model of statistical mechanics proposed by Jean-Marie Souriau. This model, referred to as Lie group thermodynamics, was developed in a series of works by Souriau [1,2,3,4,5,6,7,8,9,10,11,12,13,14,15,16,17,18,19,20].

Jean-Marie Souriau’s model—Lie group thermodynamics—constitutes a symplectic approach to statistical mechanics. This framework incorporates geometric methods into the statistical description of physical systems, wherein the Gibbs states are represented as points on a symplectic manifold, and the symmetries of the system are described by Lie groups. We offer an original interpretation of Souriau’s theory via a symplectic foliation structure generated by the coadjoint orbits of the Lie group acting on the system. Within this model, entropy is characterized as an invariant Casimir function. Transverse to this symplectic foliation—interpreted as the level sets of entropy—we associate a Riemannian foliation, corresponding to the level sets of energy. These transverse foliations define a web structure, in the sense described by Wolak [21]. We describe the dynamics along both sets of transverse leaves via a metriplectic flow, following the framework of Coquinot and Morrison [22]. This flow combines the Poisson bracket on the symplectic leaves, which describes non-dissipative phenomena while preserving entropy, with the metric flow bracket on the Riemannian leaves, which characterizes dissipative processes through entropy production.

In the context of information geometry applied to statistical manifolds, we associate the symplectic foliation with the Fisher metric and the Riemannian foliation with its dual—namely, the Hessian of the entropy function.

Jean-Marie Souriau, a French mathematician, made substantial contributions to the field of symplectic geometry by introducing pioneering concepts that deepened the understanding of geometric structures underlying dynamical systems. He explored the profound relationship between geometry, physics, and the representations of symmetry groups. Among his most significant contributions is the geometric formulation of Hamiltonian dynamical systems. Souriau formalized the idea that the dynamics of physical systems could be described geometrically on symplectic manifolds, treating trajectories of Hamiltonian systems as curves within such manifolds. He precisely characterized the action of symmetry groups on phase spaces—understood as symplectic manifolds—and demonstrated how these actions relate to system invariants. Souriau showed that symplectic geometry is intrinsically connected to the coadjoint orbits of Lie groups acting on manifolds, particularly in mechanical systems where symmetries are typically expressed via Lie groups such as the Galilean group. Crucially, Souriau recognized that contact structures—generalizing symplectic structures to odd-dimensional spaces—could be employed to describe dissipative or non-reversible dynamics. This insight allowed contact geometry to be applied to a broader class of dynamical systems, enriching the understanding of symplectic geometry, which traditionally focuses on reversible phenomena.

Souriau applied his symplectic model to both statistical mechanics and thermodynamics in his formulation of Lie group thermodynamics. This approach enables a reinterpretation of statistical mechanics and thermodynamics as natural extensions of Hamiltonian mechanics. In this view, a statistical ensemble is treated as a family of Hamiltonian systems, incorporating probability distributions on phase space. Souriau’s formulation allows the dynamics of entropy and other thermodynamic variables to emerge directly from the principles of symplectic geometry, thereby establishing a deep connection between statistical mechanics, thermodynamics, and differential geometry.

Despite its originality and depth, Souriau’s work remained largely overlooked for over fifty years. Several factors contributed to this neglect. Firstly, Souriau employed his own idiosyncratic notations, which were atypical in the context of Lie group theory, rendering his writings difficult to approach. Secondly, his principal works were written in French—including all of his papers on the subject—limiting their accessibility to a broader international audience. Thirdly, chapter 4 of his major work, which is dedicated to statistical mechanics, was often neglected by contemporary readers, many of whom were mechanicians primarily interested in the chapters focused on the geometric model of mechanics. Finally, Souriau’s theory demands a dual expertise in both thermodynamics and the representation theory of Lie groups—an intersectional knowledge base that was rare both during his time and remains so even today.

The structure of this paper is described in the following.

In Section 1, we introduce the work of the French mathematician and physicist Jean-Marie Souriau, who, after introducing symplectic geometry into the framework of classical mechanics and the calculus of variations, generalized his symplectic model for statistical mechanics. We explain how this model, which he called “Lie groups Thermodynamics”, which is part of the theory of representations of Alexandre Kirillov, allows us to reconsider the second principle of Sadi Carnot within the framework of the foliation theory introduced by Charles Erhresmann and Georges Reeb. We introduce a new, purely geometric definition of entropy, as an invariant Casimir function on the symplectic foliations, generated by the coadjoint orbits of the group, acting on the thermodynamic system. We also establish links with information geometry associated with the Fisher metric.

In Section 2, we develop this foliation model of thermodynamics, making the link with the notion of metriplectic flow, compatible with Onsager relations, describing the dynamics along each of the leaves. We show that to the symplectic foliation of Souriau, we can associate a transverse Riemannian foliation by the energy level sets, which describe the dissipative phenomena and the generation of entropy of the second principle of Sadi Carnot.

In Section 3, we recall that the physicist Baptiste Coquinot has established the compatibilities of metriplectic flow with the Onsager relations that describe dissipative phenomena. We also recall that Günther Vojta had studied first Onsager relations within the framework of symplectic geometry.

In Section 4, we give physicist Herbert B. Callen’s insight into thermodynamics as the science of symmetry. We recall avenues of study opened by Callen to explore more deeply the role of symmetry in thermodynamics.

In Section 5, we conclude with last works of Jean-Marie Souriau on thermodynamics in private unpublished documents.

We can refer to this quote by Sadi Carnot that proposes to explore thermodynamic systems for bodies in motion, illustrating the topic of our paper exploring thermodynamics for dynamical systems:

[…] The changes in temperature occurring in bodies as a result of motion have been very little studied up to now; this class of phenomena would, however, merit the attention of observers. When bodies are in motion, especially when motive power is consumed or produced, remarkable changes occur in the distribution of heat and perhaps in its quantity. We will present a small number of facts in which this phenomenon develops with the greatest evidence. [On a fort peu étudié jusqu’ici les changements de température survenus dans les corps par l’effet du mouvement; cette classe de phénomènes mériterait cependant l’attention des observateurs. Lorsque les corps sont en mouvement, lorsque surtout il se consomme ou qu’il se produit de la puissance motrice, il arrive des changements remarquables dans la distribution de la chaleur et peut-être dans sa quantité. Nous allons apporter un petit nombre de faits, où ce phénomène se développe avec le plus d’évidence.]

[23]

This second quote by Georges Reeb, the inventor of foliation structure, proves that the proposal of using foliations to interpret thermodynamic systems was in the initial ideas of the inventors:

[…] Thermodynamics has long accustomed mathematical physics [cf. DUHEM P.] to the consideration of completely integrable Pfaff forms: the elementary heat dQ [notation of thermodynamicists] representing the elementary heat given up in a reversible infinitesimal modification is such a completely integrable form. This point does not seem to have been explored much since then. [La thermodynamique a habitué de longue date la physique mathématique [cf. DUHEM P.] à la considération de formes de Pfaff complètement intégrables: la chaleur élémentaire dQ [notation des thermodynamiciens] représentant la chaleur élémentaire cédée dans une modification infinitésimale réversible est une telle forme complètement intégrable. Ce point ne semble guère avoir été creusé depuis lors.]

[24]

## 2. Jean-Marie Souriau’s Symplectic Model of Lie Group Thermodynamics and Geometric Definition of Entropy as Casimir Function on Symplectic Foliation

As observed by Charles-Michel Marle [25,26,27,28,29,30,31,32], Josiah Willard Gibbs, in chapter 4 of his book *Elementary Principles in Statistical Mechanics, developed with Especial Reference to the Rational Foundation of Thermodynamics* published in 1902 [33], considered generalization of Gibbs states built with the moment map of the product of the one-dimensional group of translations in time and the three-dimensional group of rotations in space for the study of systems contained in a rotating vessel, referring to a paper by Maxwell published in 1878. We can read the following in Gibbs’s book:

[…] The consideration of the above case of statistical equilibrium may be made the foundation of the theory of the thermodynamic equilibrium of rotating bodies, a subject which has been treated by Maxwell in his memoir On Boltzmann’s theorem on the average distribution of energy in a system of material points

[33]

Jacques Hadamard made a review of this book in 1906 [34] and wrote:

[…] This book is not one of those that one analyzes hastily; but, on the other hand, the questions it deals with have been greatly agitated in recent times; the ideas defended by Gibbs have been the subject of much controversy; the reasoning with which he supported them has also been criticized. It seems interesting to me to study his work in the light of these controversies and by discussing these criticisms

[34]

We will develop in this section Jean-Marie Souriau’s works who studied systematically thermodynamics in the case of dynamical systems under the action of a Lie group in the framework of symplectic geometry applied for statistical mechanics [1,2,3,4,5,6,7,8,9,10,11,12,13,14,15,16,17,18,19,20]. We will also make the link with the mechanics of completely integrable Hamiltonian systems (the study of complete systems of first integrals) [35], that is, in geometric language, Lagrangian foliations of symplectic manifolds [36], which was studied by Paulette Libermann under the supervision of Charles Ehresmann, as advised by Elie Cartan. Paulette Libermann observed that analysis and mechanics (and, nowadays, quantum physics) require examining the more general situation of symplectically complete foliations [37,38,39,40,41,42,43], Pang [44].

### 2.1. Souriau’s Seminal Idea of Symplectic Model of Statistical Mechanics in the Framework of Representation Theory

Jean-Marie Souriau, PhD, was supervised by André Lichnérowicz at Collège de France, who was also interested by the notion of symplectic foliation after its development by his PhD student and the work of Paulette Libermann. We can read the following in Lichnérowicz’s lecture at Collège de France:

[…] Tuesday’s class was devoted to the systematic study of the relationships between foliation and Poisson manifolds. The notion of Poisson manifold was introduced by us in 1975 as a natural contravariant generalization of that of symplectic manifold. On such a manifold, the Poisson structure determines a symplectic foliation either in a generalized sense (non-regular Poisson manifold) or in the strict sense of the term (regular Poisson manifold). A simple natural example of the first case is provided by the orbits of the coadjoint representation of a Lie algebra. A simple example of the second case is given by the fibers cotangent to the foliations. Let (M, F) be a symplectic manifold equipped with a Lagrangian foliation £. It has been shown that there always exists on M a connection adapted to foliation which induces on each leaf a flat connection without torsion. If the manifold admits a fiber-type Riemannian metric for £, it admits a Riemannian metric which induces a flat metric on each leaf. We have thus clarified and generalized recent results of A. Weinstein and P. Dazord. The same results are valid if, instead of a Lagrangian foliation, we consider an isotropic foliation of (M, F) such that the field of symplectic orthogonal planes is a coisotropic foliation.

[45]

The statistical physics community ignored Souriau’s symplectic model of thermodynamics until recently. We can only make reference to G. Vojta’s paper [46] “Symplectic Formalism for the Thermodynamics of Irreversible Processes”, making reference to Prof. Ingarden, where Votja writes the following:

[…] A characteristic trend in mathematical physics is the growing use of the same abstract formalisms for the description of very different physical phenomena. A paradigm is the Hamiltonization of various fields of physics, i.e., the use of Hamiltonian structures and symplectic geometry, based on the mathematical language of exterior differential forms, fibre bundles, Poisson bracket structures and generally Lie algebraic conceptions. Examples are widespread. With the discovery of the Lie-Poisson structure underlying the Euler equations of fluid flow by Arnol’d … Another field where Hamiltonian structures and symplectic geometry play a growing role is quantum mechanics and quantum field theory including nuclear physics. In the foreground are problems of quantization (so-called geometric quantization) by means of the Wigner-Wel formalism and the physics of semi-classical systems. A further use for Hamiltonian structures and symplectic notions is given in the fields of differential equations, optimization and control theory. Characteristic of all these theoretical developments is that the systems considered are ideal systems (fluids, plasmas, quantum systems, …) without energy dissipation (without frictions, damping, …), i.e., without entropy production. There exists a larger literature on the damping of quantized systems or, in other words, on problems of the correct formulation of a quantum theory of systems with friction. A symplectic approach to nonconservative systems- which can be considered as a first step towards a correct quantization procedure-was treated only in few papers without explicitly considering, however, the thermodynamics and, in particular, the entropy balance. On the other hand a few papers have been published on the symplectic structure of equilibrium thermodynamics, but (to the best of our knowledge) not of irreversible thermodynamics, with one important exception, i.e., a set of papers by the Ingarden group on “information geometry” and irreversible thermodynamics where indeed the connection between information theory and differential geometry plays the main role

[46]

**Figure 1:**
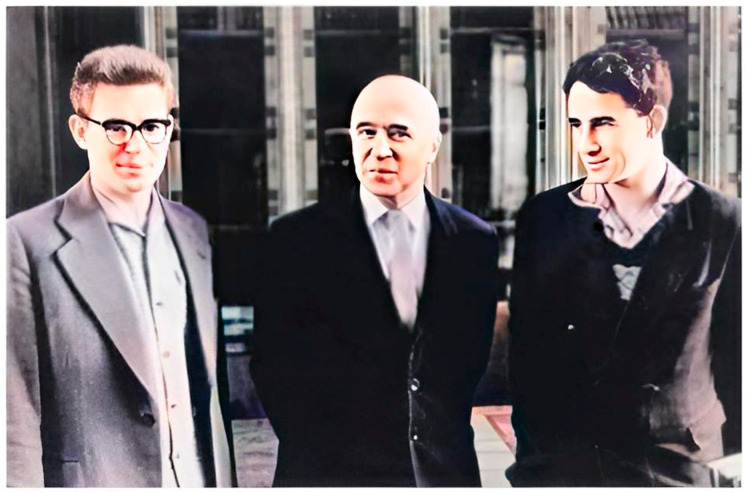
Russian school of Lie group representation theory and symplectic geometry, with Alexandre Kirillov (**on the left**), Vladimir Arnold (**on the right**), and President of Moscow State University, I. G. Petrovsky (**in the midle**). Source [49].

Lie group representation theory and symplectic geometry in mechanics were in parallel studied in Russia by Alexandre Kirillov and Vladimir Arnold [47,48] in Figure 1, but without connection with Souriau’s works on “Lie Groups Thermodynamics”. We illustrate portraits of A. Kirillov and V. Arnold as young students at Moscow State University when they have developed Lie group representation theory.

### 2.2. Jean-Marie Souriau Scientific Biography

At the end of the war, the physicist and mathematician Jean-Marie Souriau began his career as an engineer at ONERA. Influenced by presentations by American engineering researchers at ENS Paris, he was won over and made the conscious decision to do a thesis on the stability of aircraft. After his thesis supervised by André Lichnerowicz [45,50,51,52,53,54,55], he took a position at the Institute of Advanced Studies in Tunis. We will try to retrace his scientific quest for a new foundation of statistical physics, thermodynamics, and quantum mechanics on the common base of the structures of symplectic geometry. The theory of representations of Lie groups, initiated by Alexandre Kirillov [56,57], plays a central role with the notion of moment map [58], coadjoint orbits, and cohomology of Lie algebras (Nencka & Streater [59], Pavlov & Sergeev [60]) to capture the structures generated by symmetries. This quest began for Jean-Marie Souriau in the romantic setting of the ruins of Carthage, where isolated he undertook an intimate reading of the works of Lagrange and discovered the symplectic structures which had until then been hidden from the view of mechanics. On the basis of this geometric mechanics, we will discover how he had the idea of extending it to statistical mechanics and gave it the disconcerting name of “Lie Groups Thermodynamics”. As he later admitted, all this scaffolding, which he built in his solitude in Carthage and the silence of his passions, had as its ultimate goal the “geometric quantification” of quantum mechanics. The seeds of this original thought were not born by chance but in the cradle of a line of philosophers from Ecole Normale Supérieure over several generations who had shaped his spirit into the triptych “Aesthetics—Structure—Movement” and whose family work he completed in attacking the “structure of the movement”, with his uncle Etienne Souriau having transmitted to him the “structure of aestheticism” and his grandfather Paul Souriau “the Aesthetics of movement”. Reconnecting with Aristotle’s physics where movement is both a change of place (mechanics) and a change of state (thermodynamics), Jean-Marie Souriau bequeathed us a new thermodynamics based on symplectic geometry, in which through the miracles of the cohomology of Lie algebra, temperature, heat, and entropy ontologically changed their nature and acquired a purely geometric definition. The (Planck) temperature appeared as an element of the Lie algebra of the symmetry group which acts on the dynamic system, heat as an element of the dual space of the Lie algebra, and entropy as a Casimir invariant function [61] on the coadjoint orbits (orbits, themselves seen as a symplectic foliation). This last point is undoubtedly the most profound ontological rupture of the 20th century. Since Claude Shannon, entropy had only had an axiomatic definition without solid established foundations. With Souriau, entropy acquires a purely geometric archetypal definition. Consider the group which acts on the system and the associated symmetries and consider its coadjoint orbits (action of the group on the moment map; the moment map playing the role of a geometrization of Noether’s theorem), then the entropy appears clearly as the invariant Casimir function on the symplectic foliation thus created. By the same token, this symplectic foliation appears as the entropy level sets (a leaf corresponds to a constant entropy). From leaf to leaf, entropy increases as explained by the second principle of thermodynamics. This second principle thus changes its nature and is, therefore, closely linked to the structures of these symplectic foliations. Thus, a new equation allows us to characterize and “geometrically construct” the entropy starting from the symmetry group, and also allows us to rewrite the Fourier heat equation (Bachelard [62]) geometrically with a Poisson bracket (Cosserat [63]). This new vision of things also revolutionizes information theory and geometry. The Souriau model makes it possible to construct the probability density of maximum entropy for any homogeneous space on which a Lie group acts or to calculate a density associated with a group. Souriau introduced a Riemannian metric on this symplectic manifold, from what is called the KKS 2 form (Kirillov, Kostant and Souriau) and a cocycle which bears his name. We recently made the link between this Riemannian metric and the Koszul–Fisher metric of information geometry (Jean-Louis Koszul had studied this metric, in parallel with Ernest Vinberg for sharp convex cones and its invariance with respect to automorphisms of these cones). By achieving the alliance of changes of places and changes of states, the thought of Jean-Marie Souriau rediscovered the concepts of Aristotle’s physics and the epistemology of Blaise Pascal. Without a doubt, Souriau’s discovery is one of the greatest discoveries in physics of the 20th century, modifying the ontological nature of the elements of Fourier’s and Carnot’s theory of heat and giving a new status to entropy, as a fundamental archetype emerging from the cohomological structures of the symplectic foliations and the moment map associated with the dynamic group which acts on the system. Having understood that the foliation model of Souriau thermodynamics was limited to non-dissipative phenomena, we disentangled the links with non-equilibrium thermodynamics by highlighting that Riemannien foliation, transverse to symplectic foliations and the associated dynamics, corresponded to dissipative phenomena (production of entropy by passing from symplectic leaf to leaf of entropy level sets). The updated transverse structures have shown to be compatible with Onsager relations (Casimir [61], Hubmer & Titulaer [64]) and the metriplectic flow (flow coupling a Poisson bracket and a metric bracket), as proven by Baptiste Coquinot (Coquinot & Morrison [65]). The metric bracket associated with these transverse structures happens to be linked to the inverse Koszul–Fisher metric of information geometry, that is, to the Hessian of entropy. This “Souriau-ian” revolution also shakes the edifice of Claude Shannon’s information theory and Kolmogorov’s probabilities by also recasting these disciplines on the basis of symplectic geometry. The structures of probabilities and information are thus reconstructed on the basis of the theory of symplectic foliations.

**Figure 2:**
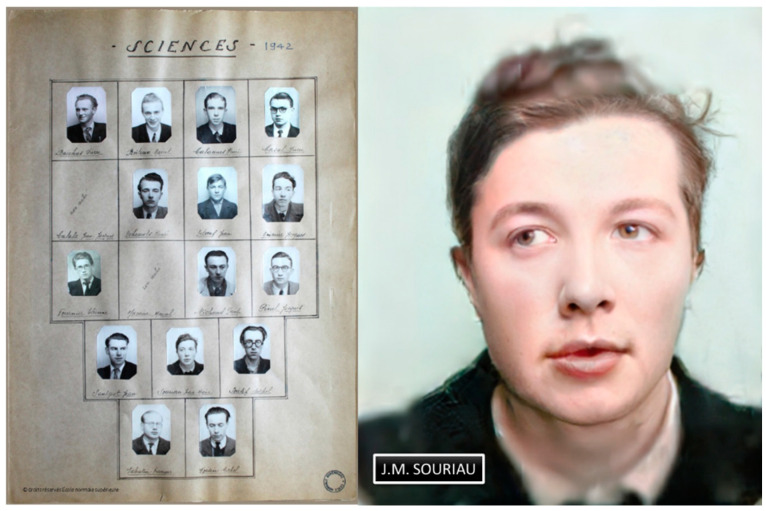
Jean-Marie Souriau, student at the Ecole Normale Supérieure in Paris in 1942, with Jacques Dixmier and René Deheuvels, among others. Source trombino ENS Paris arxiv.

Jean-Marie Souriau from 1932 to 1942 completed his secondary studies in Nancy, Nîmes, Grenoble, and Versailles, undoubtedly following the various assignments of his teaching father. Jean-Marie Souriau married Christianne Hoebrechts, who died prematurely in 1985 and with whom he had five children: Isabelle, Catherine, Yann, Jérôme, and Magali. He entered ENS Paris in 1942, passing twice in the unoccupied zone in Lyon and a second time in Paris. Also admitted to the Ecole Polytechnique, he resigned to join ENS Paris. During his studies at the ENS, he took courses at the Sorbonne with the physicist Yves Rocard and the mathematician Elie Cartan. He joined as a volunteer for La France Libre in 1944. A volunteer, he returned to the ENS in 1945 and registered for a special session of the aggregation, organized for young people who had served under the flags to liberate France, where he and his friend Gérard Debreu (awarded the Nobel Prize in economics several years later) were brilliantly received. ENS Paris students of promotion in 1942 are given in Figure 2. We include this figure to illustrate that Souriau graduated from ENS Paris at the same time as Jacques Dixmier, who developed a theory of enveloping algebra.

**Figure 3:**
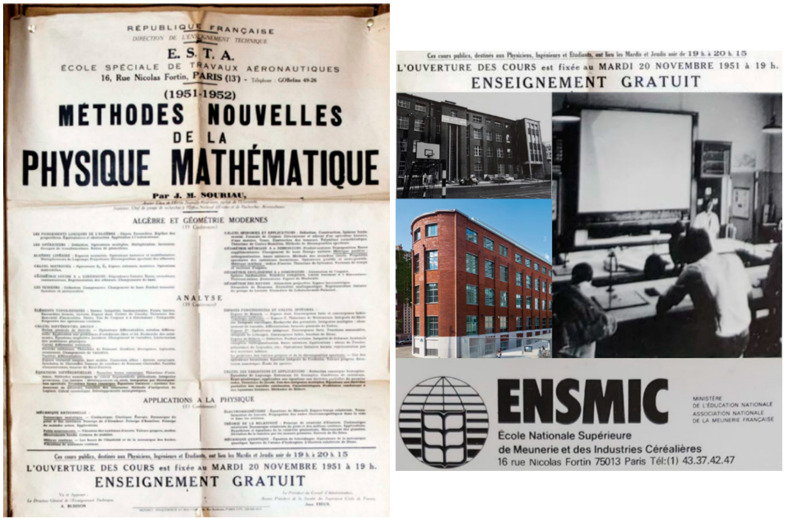
Jean-Marie Souriau’s free course at the Ecole de la Meunerie, on “New methods of mathematical physics”. Source (Vallée, de Saxcé, G. & Marle [66]).

In 1946, the same year, he joined a laboratory working on the scanning electron microscope and then joined a CNRS “theoretical physics” session as a researcher. As early as 1948, he offered a free open course, “New Methods of Mathematical Physics”, for which he twice filled the 200-seat lecture halls. Classes took place at 16 rue Nicolas Fortin, at the Ecole de la Meunerie, as illustrated in Figure 3. Souriau certainly thought of his Souriau ancestors, master millers in Vendômois. Claude Vallée testified as follows:

[…] I remember that he often told me about an evening course that he gave at the “Ecole des Meuniers” in collaboration with Jérôme Chastenet de Géry and Roger Valid (who wrote the exercises). Originally, the public courses of 1951–52 were a response to the lack of matrix calculation that Jean-Marie Souriau had felt among French aeronautical engineers, notably at the recently created ONERA. The simple addition of 2 matrices was respectfully considered a very abstract notion. These shortcomings prevented engineers from understanding the progress made by American aircraft manufacturers during the Second World War (progress that allowed them to win the air war while the English won the sea war) … This is what motivated, in the 1950s, the need for a good understanding of matrix calculation to master “Structural Mechanics” and design prototypes of new aircraft…conferences on “Modern Algebra and Geometry” summarized on the poster.

(Vallée, de Saxcé, G. & Marle [66])

Souriau made this course into a “Linear Calculation” book. There, we can find a presentation of the calculation of the parameters of the characteristic polynomial of a matrix, improving the method of Urbain Jean Joseph Leverrier of 1840. As early as 1955, Souriau’s algorithm was tested and compared by the National Bureau of Standards of Los Angeles, under the sponsorship of the Wright Air Development Center, the US Air Force, and the Office of Naval Research, and was concluded at the University of California, by the Office of Naval Research.

He finally opted for a career as an aeronautical engineer at ONERA, becoming head of research teams and defending his thesis in June 1952 on the theme of “aircraft stability” under the direction of André Lichnerowicz (professor at Collège de France) and Joseph Pérès (collaborator of Vito Volterra); this thesis was used to design the “Caravelle” and “Concorde” aircraft (ONERA obtains royalties on the Souriau patents). In this thesis, he refers to the work of Yves Rocard on “General dynamics of vibrations”. In his thesis, he writes the following:

[…] I studied the problems of vibrations and stability which arise in aeronautics and in some other techniques; this work allowed me to develop stability criteria which are presented in the form of algorithms that can be easily calculated from theoretical or test data; they have since been regularly used in various fields (subsonic and supersonic aircraft, navigation instruments, etc.).

Souriau [1]

In an interview, Souriau speaks in these terms about the content of his thesis:

[…] We couple the elastic properties of the wings of an airplane with the dynamics of the atmosphere described by partial differential equations and a sheet of vortex discontinuities. With all this, we calculate a complex determinant and we count how many turns it makes around the origin when a pulsation ω varies. If it makes the right number of revolutions, the plane is stable; otherwise it will start to vibrate and explode. And it works! It was used for planes like the Concorde. The result was that we could put the motors anywhere, and it made no difference to stability. As a result, we started to put the engines on the rear tail and for 25 years, all the planes that had engines at the rear paid royalties to France, but not to me.

Souriau [1]

**Figure 4:**
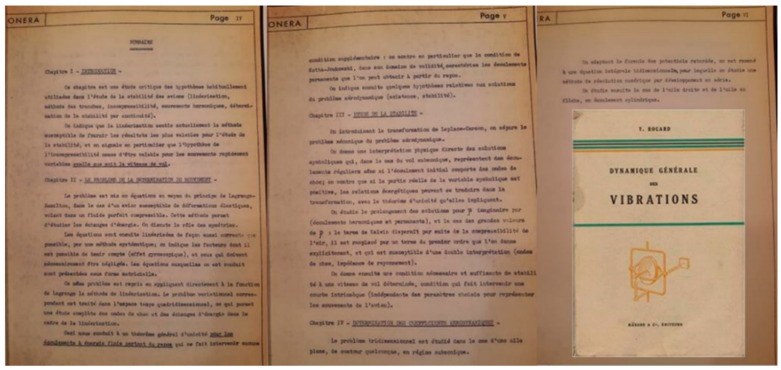
Pages from Jean-Marie Souriau’s thesis, “On the stability of aircraft”, defended on 20 June 1952, including the bibliographic page which refers to the work of Yves Rocard. *Source* personal photo of Souriau’s thesis manuscript archived at the ONERA.

I obtained a copy of this thesis through colleagues at ONERA (Vincent Brion and Agnès Dolfi-Bouteyre), the cover and certain pages of which I reproduce in Figure 4.

Professor Jean-Pierre François wrote the following in “Dynamic Systems applied to Oscillations” (Françoise [67]) about Jean-Marie Souriau’s thesis:

[…] One of the most important contributions of the theory of dynamic systems to applications is the study of stability. It is not always very easy in a concrete situation to put this study into practice. The thesis of J.-M. Souriau is a beautiful illustration of this with a very delicate discussion of the possible hypotheses in the study of the stability of aircraft, the choice of a linearization method and the mathematical solution proposed in the form of calculation of a complex determinant for which we calculate the number of turns it makes around the origin. In the framework of the theory of systems on several time scales, new stability problems arise. For example, with the theory of dynamic bifurcations introduced by R. Thom, we can discuss delays at bifurcation. The orbits corresponding to the maximum delays (maximum canards) are now considered as “separators” beyond which we observe a very rapid transition towards new attractors.

(Françoise [67])

During this period, in 1948, he also invented an algorithm called the Leverrier–Souriau algorithm which allows the characteristic polynomial of a matrix to be calculated and was used on the first IBM computers. From 1948 to 1952, he also provided continuing education at the Special School of Aeronautical Works (ESTA, Paris) under the general title “New methods of mathematical physics”. From 1951 to 1952, he created and ran the mechanics course in the third year of the École Normale Supérieure de l’Enseignement Technique (ÉNSET, Paris). From 1952, he also had university training in the following disciplines: mathematics, mechanics, relativity, mathematical methods of physics, and computer science.

**Figure 5:**
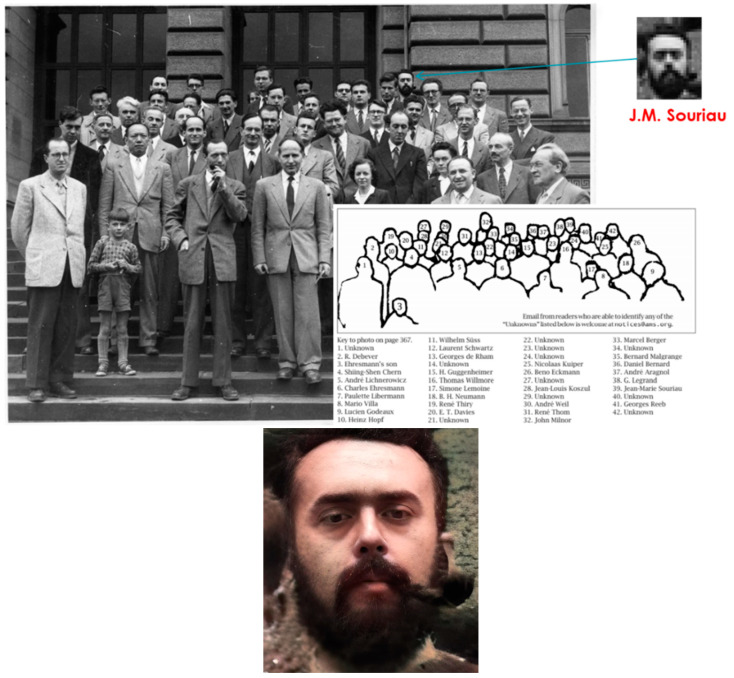
Jean-Marie Souriau at the “Differential Geometry” Conference in Strasbourg in 1953. The same photograph includes Jean-Louis Koszul, André Weil, Shiing-Shen Chern, Georges de Rham, Charles Ehresmann, Lucien Godeaux, Heinz Hopf, André Lichnerowicz (thesis director of Jean-Marie Souriau), Bernard Malgrange, John Milnor, Georges Reeb, Laurent Schwartz, René Thom, and Paulette Libermann. *Source* (Audin [69]).

After his thesis in 1952, he joined the Institut des Hautes Etudes, rue de Rome in Tunis, and settled with his wife in Carthage. It was during this period that he reread and deepened the work of Lagrange in analytical mechanics and discovered the symplectic structures that he formalized in his work “Structure of dynamic systems”. It was while thinking about his exchanges with ONERA engineers that he invented his masterpiece, the “moment map”, a geometrization of Noether’s theorem (Noether [68]) (the components of the moment map are the invariants of Noether). In 1953, he was invited, with another young researcher named Jean-Louis Koszul, to the famous conference in Strasbourg on “differential geometry” organized by his PhD supervisor, André Lichnérowicz, as illustrated in Figure 5.

We can read the following in the interview by Patrick Iglesias:

[…] It was with the memory of discussions with engineers who asked the following question: what is essential in mechanics. I remember very well an engineer asking me: is mechanics simply the principle of conservation of energy? This is good for a one-parameter system, but once there are two, it’s not enough. I had of course learned the Lagrange equations and all the analytical principles of mechanics, but it was all just a recipe book; we have not seen real principles.

(Souriau [15])

He remained in Tunis from 1952 to 1958, as a lecturer, then as a full professor at the Institut des Hautes Études. In 1953, he participated in the CNRS conference on differential geometry in Strasbourg, as illustrated in Figure 5, and he published his first work there based on his work begun in Carthage, entitled “Differential symplectic geometry. Applications”. Charles Ehresmann and André Lichnerowicz, the organizers of this 1953 conference, stated in the introduction to the proceedings: “We have especially endeavored to highlight some of the new paths in which our science is embarking. We also wanted young mathematicians to be able to highlight their thoughts and their results”.

In 1956, he created the International Conference on Variational Theories (CITV) which is held every year for high-level research on the themes he initiated. This conference was renewed in 1996 with the help of Claude Vallée from the University of Montpellier and from 2012 by Gery de Saxcé from the University of Lille (de Saxcé & Vallée [70,71,72,73,74]). The last CITV’23 conference was organized in Porquerolles.

In 1958, he became a professor at the University of Aix-Marseille. He remained in Marseille throughout his career and from 1978 to 1985 became director of the Marseille Center for Theoretical Physics (CNRS laboratory) in charge of the theoretical mechanics, geometry and quantification, astronomy, and cosmology teams. He was also a professor of mathematics at the University of Provence (Aix-Marseille I) and ended up as an exceptional second-level professor. For five years, he was the director of the third inter-university cycle of pure mathematics in Marseille and for five years the third inter-university cycle of theoretical physics in Marseille-Nice.

In 1974, Souriau organized the international conference “Symplectic Geometry and Mathematical Physics” in Aix-en-Provence, and he wrote the proceedings, published in a new bound volume under this title in 1975 by Editions du Center National de la Recherche Scientifique. Souriau’s contribution to the proceedings is a 65-page article “Statistical mechanics, Lie groups and cosmology” which begins with the chapter “Symplectic formulation of statistical mechanics” which summarizes and extends chapter 4, “Statistical mechanics”, of his fundamental book *Structure of dynamic systems* (Souriau [7]), published in 1970 (copyright 1969, legal deposit October 1969). It is in this article that he develops what he called “Lie groups Thermodynamics” which is a symplectic model of statistical mechanics, where the temperature (of Planck) is generalized to all invariants (not only the energy, but also the angular moments) and takes on a geometric meaning as an element of the Lie algebra of the group acting on the system. In the introduction to the volume of proceedings, Souriau writes that symplectic geometry is not, strictly speaking, a new theory, but it comes from the work of Lagrange in 1788 [75] of which Souriau gave a modernized formulation. Souriau further wrote that applications of symplectic geometry had become numerous, touching “a very wide range of subjects”. Souriau developed the fact that Lagrange had been at the origin of these structures, and he wrote an article in 1986 on this subject titled “The symplectic structure of mechanics described by Lagrange in 1811” (Souriau [13]), published in issue No. 94 of *Mathematics and Human Sciences*.

He was also a member of the French Mathematical Society (SMF) and the French Society of Astronomy Specialists. For five years, he also taught courses in the third inter-university cycle of pure mathematics in Marseille and the third inter-university cycle of theoretical physics in Marseille-Nice. He was a member of the editorial board of the *Journal of Geometry and Physics* in Florence. He organized two international CNRS conferences in 1968 and 1981 and the Days of the Mathematical Society of France. He was one of the main actors in the creation of the Center for Theoretical Physics of Luminy, which he directed from 1978 to 1985. Honored with the Academic Palms and the National Order of Merit, he obtained the prize on the theme “Vibrations” in competition by the Association for Aeronautical Research in 1952 and the prize on the theme “Cosmology” in competition by the Association for Aeronautical Research in 1952. Other awards include the Louis Jacot Foundation in 1978, Grand Prix Jaffe of the Academy of Sciences in 1981, and Grand Prix Scientifique de la Ville de Paris in 1986. Jean-Marie Souriau died in 2012 at the age of 90.

In 2019, a conference was organized at Paris-Cité University and the Henri Poincaré Institute, with the title “Souriau 2019”. We can note the keynote by Jean-Pierre Bourguignon (former director of IHES and the European ERC), “Jean-Marie Souriau and Symplectic Geometry” (Bourguignon [76]). Bourguignon recounts that as a young student at the Ecole Polytechnique, they had initiated a seminar between students to compensate for the low level of mechanics courses. It was at this time that they came across Souriau’s book which fascinated them by the fact that Souriau reintroduced analytical mechanics on the solid foundations of symplectic geometry and the calculus of variations. Bourguignon had the opportunity several times to meet and speak to Souriau in the conferences he organized.

### 2.3. Jean-Marie Souriau Elaboration of Symplectic Model of Mechanics and Lie Group Thermodynamics

Souriau’s symplectic idea germinated on the ruins of Carthage and in the reading of the works of Lagrange. Souriau specifies the following in an interview:

[…] In 1952, I left everything and went to the University of Tunis… The way the administration understood research. You had to search for so many hours a day. There were little windows in the doors so the guards could see if we were doing math or not. I have a friend who was fired for political reasons... This period played a big role in my life, for personal reasons. From a research point of view I began to meditate on the practice of mechanics. When you invert a three by three size matrix, you see a denominator common to all the terms appear, you have discovered the determinant. Having noticed that strange antisymmetric things appeared in the equations of mechanics, I said to myself: this is exactly like Euclidean spaces except that it is quite the opposite. I thus had the idea of doing differential symplectic geometry, the title of my first work published on this subject in 1953… It was much later that I understood that it was implicit in Lagrange. The essential idea is that the solutions of the equations of motion of a dynamic system constitute a symplectic manifold. And I thought that there was an interest in studying this type of manifold, just as there is an interest in studying Riemannian manifolds... It was with the memory of discussions with engineers who asked the following question: what is what is essential in mechanics. I remember very well an engineer who asked me: is mechanics simply the principle of conservation of energy? This is fine for a one-parameter system, but as soon as there are two, it’s not enough. I had of course learned the Lagrange equations and all the analytical principles of mechanics, but all that was a recipe book; we did not see any real principles there.

(Souriau [15])

**Figure 6:**
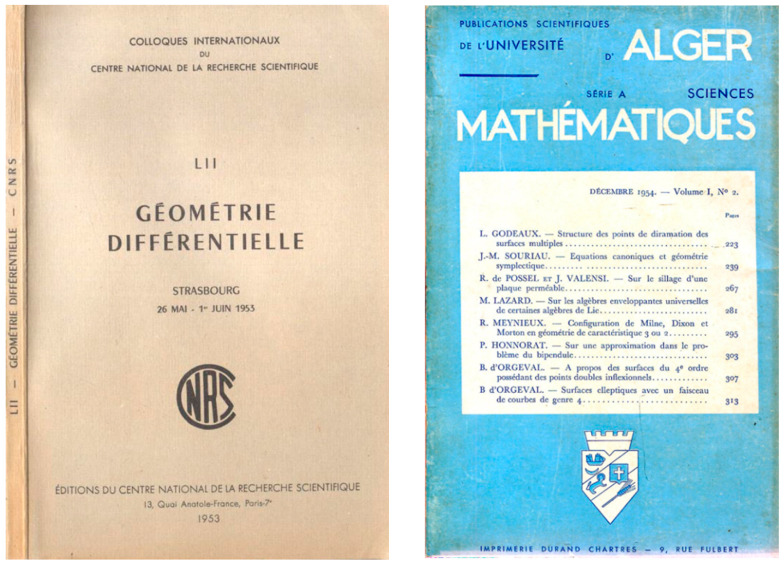
The founding articles of symplectic geometry by Jean-Marie Souriau written in Carthage and published in 1953 as “Differential symplectic geometry” in the proceedings of the Strasbourg conference and in 1954 as “Canonical equations and symplectic geometry” in the scientific publication of the University of Algiers. *Source* Gallica.

Seminal work of Jean-Marie Souriau was published in 1953 in the proceedings of Strasbourg conference on “Differentiual Geomtry” and in an Algerian scientific publication on mathematics, as illustrated in Figure 6.

Souriau explains in this same interview the genesis of this symplectic revolution:

[…] In my first publication, there was also the word “application”. I applied this formalism to the calculation of disturbances, introducing saturated isotropic manifolds (which today we call Lagrangian manifolds) which make it possible to produce so many symplectomorphisms, while there are so few “riemannomorphisms”. Earlier I was talking about determinants which appear miraculously when we try to invert a matrix. For symplectic geometry it’s a bit the same thing. You try to resolve the disturbances of a system and you see the coefficients of the symplectic structure appear. You want to solve a problem, you solve it by hand, you work, and when you have worked well, you see something appear that was hidden underneath. And what Lagrange saw, which Laplace did not see, was the symplectic structure. Finally, if you look closely at the progression of mathematics, you realize that it is very often like that. It’s usage that tells you if it’s important, and then you axiomatize things. But that comes after the fact. What makes symplectic geometry important is that it is self-imposed. I am not a Platonist, I am not saying that mathematical ideas are ready-made and that we only have to discover them. We discover physics. Symplectic geometry was discovered as a tool for celestial mechanics. Starting from a general theory of differential equations, we would probably never have found it. The particular model of the equations of celestial mechanics was richer than the model of “general” differential equations… What makes the theory global, and therefore geometric, is the action of groups of symplectomorphisms. Think of the theorem of Noether, a mathematician at the origin of an important part of modern algebra, but who also discovered this theorem which teaches us that the symmetries of a system lead to conserved quantities. It hides (or reveals) the relationships between group and symplectic. I implemented something that I thought was new, but which had existed since Sophus Lie, a geometrization of Noether’s theorem. I called it “moment map”. The initial variational formulation has exceptions which disappear with the symplectic formulation.

(Souriau [15])

In fact, very quickly, Souriau, back in Marseille, realized that his symplectic model was more suited to quantum physics than to classical analytical mechanics. The final chapters of his book would be devoted to quantum mechanics. Until the end of the 1990s, he built his final edifice on “geometric quantification”. His statistical mechanics was also a step towards quantum mechanics. On this subject, Souriau specifies the following:

[…] In 1958, I returned to France, to Marseille. And there I found myself confronted with theoretical physicists and the problems of quantum mechanics which had disturbed me during my studies like all students, I think. I realized that symplectic geometry was an essential tool for quantum mechanics. And that in fact it was even more appropriate for quantum mechanics than it was for classical mechanics. When I wrote my book on the subject I wanted to write a book on quantum mechanics and I realized that I had to present all classical mechanics in detail, as well as statistical mechanics. These were not foreign theories since they were linked by symplectic structure and symmetries. You take two particles which revolve around each other following Newton’s laws, and then you take a hydrogen atom of which you only see the spectrum. These are two objects which a priori have nothing to do with each other; but they have symplectic symmetries in common. A door is ajar.

(Souriau [15])

Jean-Marie Souriau introduced the terminology “symplectic geometry”. His first work on the subject, entitled “Differential Symplectic Geometry. Applications”, was presented at the CNRS conference in Strasbourg in 1953. In 1954, he summarized all the details of his presentation given in Strasbourg in the article “Canonical equations and symplectic geometry” in a scientific publication of the University of Algiers.

We will start by recalling Souriau’s approach to analytical mechanics by introducing symplectic structures and then explain how he applied this symplectic model to statistical mechanics.

By exploring Lagrange in Carthage, Souriau discovered that behind the Lagrangian approach (Lagrange’s brackets) was a symplectic structure, which he revealed in his book *Structure of dynamic systems* in 1969. To this end, he proposed a rewriting of the equations of mechanics classically given in phase space $\left(\begin{array}{c} r\\ v \end{array}\right)$:

(1) $$m\frac{d^{2}r}{dt^{2}}=F\;\Rightarrow m\frac{dv}{dt}=F\;\mathrm{and}\;v=\frac{dr}{dt}$$

Souriau rediscovers that Lagrange considered the space of movements $y=\left(\begin{array}{c} t\\ r\\ v \end{array}\right)\in V$:

(2) $$\left\{\begin{array}{l} m\delta v-F\delta t=0\\ \delta r-v\delta t=0 \end{array}\right.$$

This system of equations describes all of the solutions of the dynamic system which is then represented by a foliation, which represents all of the solutions to these equations. Indeed, classical mechanics is interested in one solution, while foliation theory will consider all possible solutions whatever the initial conditions.

This foliation is given by an anti-symmetric second order σ covariant tensor, called the Lagrange(–Souriau) form, a bilinear operator on the tangent vectors to V (space of movements)

(3) $$\begin{array}{l} \sigma \left(\delta y\right)\left(\delta^{\prime }y\right)=\langle m\delta v-F\delta t,\delta^{\prime }r-v\delta^{\prime }t\rangle -\langle m\delta^{\prime }v-F\delta^{\prime }t,\delta r-v\delta t\rangle \\ \mathrm{with}\;\delta y=\left(\begin{array}{c} \delta t\\ \delta r\\ \delta v \end{array}\right)\;\mathrm{and}\;\delta^{\prime }y=\left(\begin{array}{c} \delta^{\prime }t\\ \delta^{\prime }r\\ \delta^{\prime }v \end{array}\right) \end{array}$$

In the Souriau–Lagrange model, σ is a two-form on the evolution space V, and the differential equation of motion implies δy∈ε

(4) $$\begin{array}{l} \sigma \left(\delta y\right)\left(\delta^{\prime }y\right)=0\;,\;\forall \delta^{\prime }y\\ \sigma \left(\delta y\right)=0\;\mathrm{where}\;\delta y\in \ker\left(\sigma \right) \end{array}$$

In his work, Souriau only refers to a single author to free himself from the coordinate system, the thesis of François Gallissot from 1954 [77] and in particular to the following Gallissot theorem:

**Gallissot’s theorem**: There are three types of differential forms generating the equations of motion of the material point, invariant under the action of the Galileo group:

(5) $$\begin{array}{l} A:\;\left\{\begin{array}{l} s=\frac{1}{2m}\sum_{i=1}^{3}{\left(mdv_{i}-F_{i}dt\right)}^{2}\\ e=\frac{m}{2}\sum_{j=1}^{3}{\left(dr_{j}-v_{j}dt\right)}^{2} \end{array}\right.\\ B:\;f=\sum_{1}^{3}\delta_{ij}\left(dr_{i}-v_{i}dt\right)\left(mdv_{j}-F_{j}dt\right)\;\mathrm{with}\;\delta_{ij}\;\mathrm{krönecker}\;\mathrm{notation}\\ C:\;\omega =\sum_{1}^{3}\delta_{ij}\left(mdv_{i}-F_{i}dt\right)\wedge \left(dr_{j}-v_{j}dt\right) \end{array}$$

dω=0 constrains the Pfaff form $\delta_{ij}F_{i}dx_{j}$ to be closed and to be reduced to the differential of U:

(6) $$C\Rightarrow \omega =m\delta_{ij}dv_{i}\wedge dr_{j}-dH\wedge dt\;\mathrm{with}\;H=T-U\;\mathrm{and}\;T=1/2\sum_{i=1}^{3}m{\left(v_{i}\right)}^{2}$$

This proves that ω has an exterior differential dω generating the Poincaré–Cartan integral invariant:

(7) $$d\omega =\sum_{i=1}^{3}mv_{i}dr_{j}-Hdt$$

**Figure 7:**
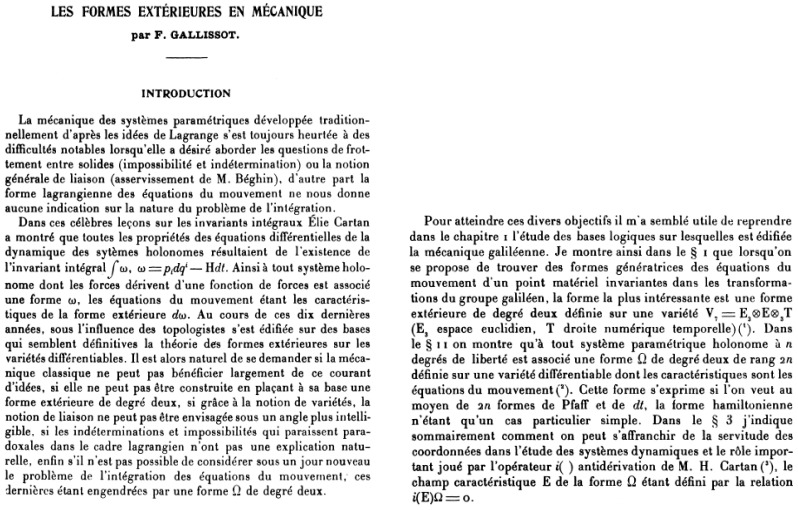
Thesis by François Gallissot defended in Chartres, as main inspiration of Jean-Marie Souriau (Gallissot [77] in 1952). *Source* NUMDAM.

The publication of François Gallisot is given in Figure 7.

### 2.4. Souriau’s Lie Group Thermodynamics as Symplectic Model of Statstical Mechanics

In chapter 4 of his book *Structure of dynamic systems*, Jean-Marie Souriau extends his symplectic model to statistical mechanics and introduces a “Lie Groups Thermodynamics”. Based on this model, we will show a geometric characterization of entropy as a Casimir invariant function in coadjoint representation, where the Souriau cocycle is a measure of the absence of equivariance of the moment map. The dual space of the Lie algebra realizes a foliation via the coadjoint orbits (Coleman [78]); symplectic leaves are also the level sets of entropy. In the context of thermodynamics, the dynamics along these foliations describe non-dissipative phenomena, while the dynamics transverse to these symplectic leaves are representative of dissipative phenomena. The model also establishes the second principle of thermodynamics, linked to the definite positivity of the tensor associated with the generalization of the Koszul–Fisher metric of information geometry. We reveal a new geometric Fourier heat equation, which is written intrinsically as an Euler–Poincaré equation. The Souriau entropy as a Casimir function is thus characterized by the Poisson cohomology introduced by Jean-Louis Koszul.

**Figure 8:**
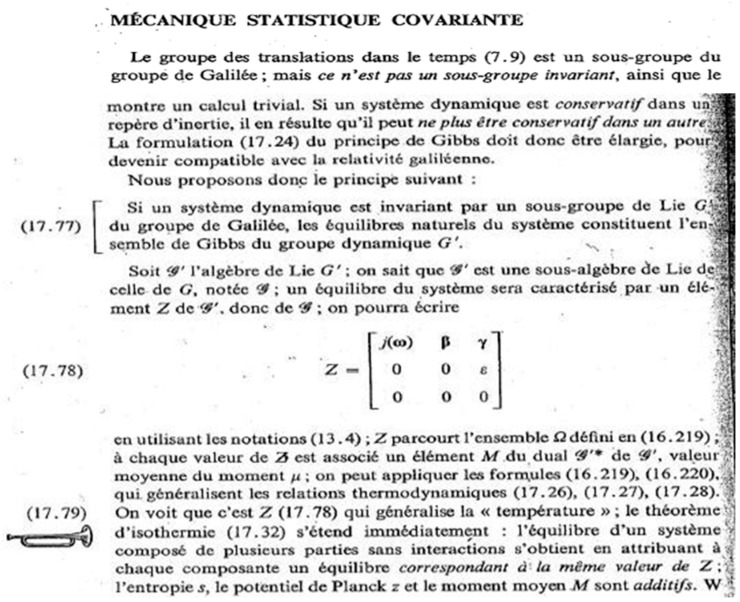
Covariant statistical mechanics by Jean-Marie Souriau in his book. *Source* (Souriau [7]).

In this “Lie groups Thermodynamics”, Souriau highlights the following facts as illustrated in Figure 8:

- The geometric temperature (of Planck) is an element of the Lie algebra of the dynamic group (Galileo group for classical mechanics or Poincaré group for relativistic mechanics for example) acting on the system and the geometric heat an element of the dual of the Lie algebra.
- Geometric entropy is the Legendre transform of the opposite of the logarithm of the Laplace transform.
- The Fisher metric of information geometry is associated with the KKS (Kostant–Kirillov–Souriau) two-form, with the two-form conferring a symplectic structure to the coadjoint orbits associated with the moment map.
- The Fisher metric is identified with a geometric heat capacity (Hessian of the Massieu potential).
- Entropy is an invariant Casimir function along the symplectic leaves, which are themselves given by the coadjoint orbits (action of the group on the moment map).

In the following section, we will use these notations and definitions:

- Lie and dual Lie algebras:
  (8) $$\mathrm{Lie}\;\mathrm{algebra}:g=T_{e}G:$$
  Dual space of Lie algebra $g^{*}$
- Coadjoint operator:
  (9) $$\begin{array}{l} Ad_{g}^{*}={\left(Ad_{g^{-1}}\right)}^{*}\\ \mathrm{with}\;\langle Ad_{g}^{*}F,Y\rangle =\langle F,Ad_{g^{-1}}Y\rangle ,\forall g\in G,Y\in g,F\in g^{*} \end{array}$$
- Moment map:
  (10) $$J(x):M\to g^{*}\;\mathrm{such}\;\mathrm{that}\;J_{X}(x)=\langle J(x),X\rangle ,\;X\in g$$
- Souriau one-cocycle:
  (11) $$\theta \left(g\right)$$
- Souriau two-cocycle:
  (12) $$\tilde{\Theta }\left(X,Y\right)=J_{\left[X,Y\right]}-\left\{J_{X},J_{Y}\right\}$$
  where
  (13) $$\begin{array}{l} g\times g\to R\\ X,Y\mapsto \tilde{\Theta }\left(X,Y\right)=\langle \Theta (X),Y\rangle \;\mathrm{with}\;\Theta (X)=T_{e}\theta \left(X(e)\right) \end{array}$$
- Affine coadjoint operator:
  (14) $$Ad_{g}^{\#}(.)=Ad_{g}^{*}(.)+\theta \left(g\right)$$
- Poisson bracket:
  (15) $$\left\{F,G\right\}\left(X\right)=\langle X,\left[\frac{\partial F}{\partial X},\frac{\partial G}{\partial X}\right]\rangle$$
- Affine Poisson bracket with cocycle:
  (16) $${\left\{F,G\right\}}_{\tilde{\Theta }}\left(X\right)=\langle X,\left[\frac{\partial F}{\partial X},\frac{\partial G}{\partial X}\right]\rangle +\langle \Theta \left(\frac{\partial F}{\partial X}\right),\frac{\partial G}{\partial X}\rangle$$
- Foliation: A foliation can be thought of as a structure where one “cuts” the manifold into a set of smooth leaves (submanifolds), and the overall structure of the foliation can be very different from simply cutting the manifold into disjointed pieces. The leaves can “bend” or “twist” across the manifold in a regular way. The concept of foliation is particularly used in geometric, topological, and analytical studies and appears in many areas, including dynamics, geometry of manifolds, and physics (e.g., in models of dynamical systems or phase structures).
- Lie algebra cohomology: Lie algebra cohomology can be seen through a geometric interpretation. For example, in differential geometry, Lie algebra cohomology appears in the study of local symmetries of a manifold, connection structures on bundles, and complexes of differential forms associated with Lie algebras. Lie algebra cohomology is a way to measure the obstructions to the possibility of “deforming” a structure given by a Lie algebra. It allows for the study properties of Lie algebras, such as representations and internal structure, in a very general and abstract way. We will use the default of cohomology, where a cocycle appears when coadjoint operator is not equivariant.

We introduce entropy into Souriau’s model of “Lie groups Thermodynamics”, a symplectic model of statistical physics. The entropy $S\left(Q\right)$ is defined on the coadjoint affine orbit of the Lie group that acts (where Q is a “geometric” heat, element of $g^{*}$ the dual space of the Lie algebra of the group). It has an invariance property $S\left(Ad_{g}^{\#}(Q)\right)=S\left(Q\right)$ if we note the affine coadjoint action $Ad_{g}^{\#}(Q)=Ad_{g}^{*}(Q)+\theta \left(g\right)$ where $\theta \left(g\right)$ is called the Souriau cocycle and is associated with the lack of equivariance of the moment map (Barbaresco [79,80,81,82,83,84,85,86,87,88,89,90,91,92,93,94,95]). In the framework of Souriau’s Lie group thermodynamics, we then characterize the entropy as a Casimir invariant function in coadjoint representation. When *M* is a Poisson manifold, a function on *M* is a Casimir function if and only if this function is constant on each symplectic leaf (the non-empty open subsets of the symplectic leaves are the smallest embedded manifolds of *M* which are submanifolds of Poisson). Classically, entropy is defined axiomatically as Shannon or von Neumann entropy without geometric considerations. In the Souriau model, the entropy will be characterized as a solution to the Casimir equation given for the following affine equivariance:

(17) $${\left(ad_{\frac{\partial S}{\partial Q}}^{*}Q\right)}_{j}+\Theta {\left(\frac{\partial S}{\partial Q}\right)}_{j}=C_{ij}^{k}ad_{{\left(\frac{\partial S}{\partial Q}\right)}^{i}}^{*}Q_{k}+\Theta_{j}=0$$

where $\Theta (X)=T_{e}\theta \left(X(e)\right)\;\mathrm{with}\;\tilde{\Theta }\left(X,Y\right)=\langle \Theta (X),Y\rangle =J_{\left[X,Y\right]}-\left\{J_{X},J_{Y}\right\}$ by noting J the moment map, and $\theta \left(g\right)$ the symplectic Souriau cocycle, which appears in the case with non-zero cohomology (i.e., in the case of non-equivariance of the coadjoint operator on the moment map).

The KKS (Kostant–Kirillov–Souriau) two-form which associates a homogeneous symplectic manifold structure with coadjoint orbits will be linked to the extension of the Koszul–Fisher metric of information geometry. The symplectic leaves associated with these coadjoint orbits are the level sets of entropy. In the context of thermodynamics, we interpret that the dynamics on these symplectic leaves describe non-dissipative phenomena, while the dynamics transverse to these leaves describe the dissipative phenomena.

The dynamics is given by the following:

(18) $$\frac{dQ}{dt}=ad_{\frac{\partial H}{\partial Q}}^{*}Q+\Theta \left(\frac{\partial H}{\partial Q}\right)$$

with the equilibrium when

(19) $$H=S\Rightarrow \frac{dQ}{dt}=ad_{\frac{\partial S}{\partial Q}}^{*}Q+\Theta \left(\frac{\partial S}{\partial Q}\right)=0$$

The numerical scheme associated with this flow preserves the coadjoint orbits and the Casimir functions of the Lie–Poisson equation.

We will also observe that

(20) $$dS={\tilde{\Theta }}_{\beta }\left(\frac{\partial H}{\partial Q},\frac{\partial S}{\partial Q}\right)dt\mathrm{where}\;{\tilde{\Theta }}_{\beta }\left(\frac{\partial H}{\partial Q},\frac{\partial S}{\partial Q}\right)=\tilde{\Theta }\left(\frac{\partial H}{\partial Q},\frac{\partial S}{\partial Q}\right)+\langle Q,\left[\frac{\partial H}{\partial Q},\frac{\partial S}{\partial Q}\right]\;\rangle$$

which founds the second principle of thermodynamics by the defined positivity of the Souriau tensor ${\tilde{\Theta }}_{\beta }\left(.,.\right)$ linked to Fisher information or to the extension of the second Koszul form of information geometry.

In the context of information geometry, the Riemannian metric of an exponential family is given by the Fisher information matrix defined by the following:

(21) $$g_{ij}=-{\left[\frac{\partial^{2}\Phi }{\partial \theta_{i}\partial \theta_{j}}\right]}_{ij}\;\mathrm{with}\;\Phi (\theta )=-\log\int_{R}e^{-\langle \theta ,y\rangle }d\lambda (y)=-\log\psi_{\Omega }$$

**Figure 9:**
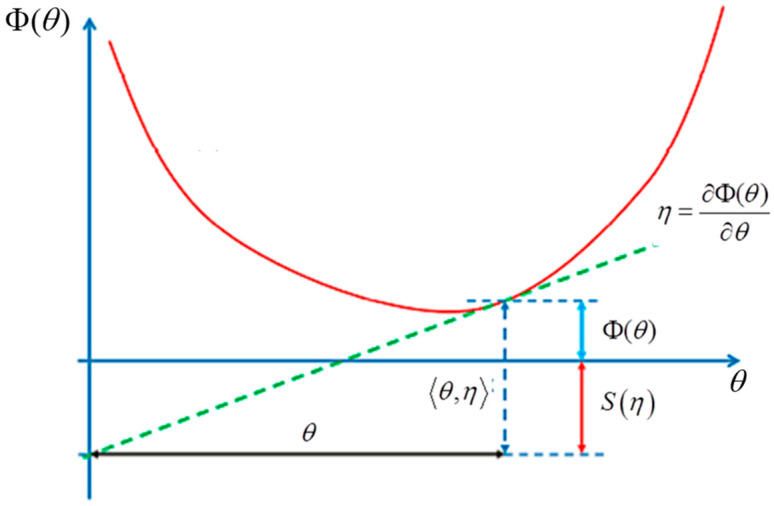
Legendre transform $S(\eta )=\langle \theta ,\eta \rangle -\Phi (\theta )$ between entropy S(η) and Massieu characteristic function Φ(θ). Curve in red Φ(θ) is a convex function on which we can define a tangent vector $\eta =\frac{\partial \Phi (\theta )}{\partial \theta }$ that intersects the vertical axis and defines value S(η).

The Shannon entropy is given by the Legendre transform, as illustrated in Figure 9:

(22) $$S(\eta )=\langle \theta ,\eta \rangle -\Phi (\theta )\;\mathrm{with}\;\eta_{i}=\frac{\partial \Phi (\theta )}{\partial \theta_{i}}\;\mathrm{and}\;\theta_{i}=\frac{\partial S(\eta )}{\partial \eta_{i}}$$

$\Phi (\theta )=-\log\int_{R}e^{-\langle \theta ,y\rangle }d\lambda (y)$ is linked to the generating function of cumulants in statistics.

In the Souriau model, the structure of the information geometry is preserved and extended over the symplectic manifold *M* associated with the coadjoint orbits:

(23) $$I(\beta )=-\frac{\partial^{2}\Phi }{\partial \beta^{2}}\;\mathrm{and}\;\Phi (\beta )=-\log\int_{M}e^{-\langle \beta ,U(\xi )\rangle }d\lambda \;\mathrm{with}\;U:M\to g^{*}$$

(24) $$S(Q)=\langle \beta ,Q\rangle -\Phi (\beta )\;\mathrm{with}\;Q=\frac{\partial \Phi (\beta )}{\partial \beta }\in g^{*}\;\mathrm{and}\;\beta =\frac{\partial S(Q)}{\partial Q}\in g$$

In Souriau’s thermodynamic model of Lie groups, β is a “geometric” (Planck) temperature, element of Lie algebra g of the group, and Q is a “geometric” heat, element of the dual space of the Lie algebra $g^{*}$ of the group. Souriau proposed a Riemannian metric that we identified as a generalization of Koszul–Fisher’s metric:

(25) $$I\left(\beta \right)=\left[g_{\beta }\right]\;\mathrm{with}\;g_{\beta }\left(\left[\beta ,Z_{1}\right],\left[\beta ,Z_{2}\right]\right)={\tilde{\Theta }}_{\beta }\left(Z_{1},\left[\beta ,Z_{2}\right]\right)$$

(26) $${\tilde{\Theta }}_{\beta }\left(Z_{1},Z_{2}\right)=\tilde{\Theta }\left(Z_{1},Z_{2}\right)+\langle Q,ad_{Z_{1}}(Z_{2})\;\rangle \;\mathrm{where}\;ad_{Z_{1}}(Z_{2})=\left[Z_{1},Z_{2}\right]$$

Souriau’s fundamental theorem is that “Any symplectic manifold on which a Lie group acts transitively by a Hamiltonian action is a space covering a coadjoint orbit”. We can observe that the Fisher metric is this non-equivariant case two-form extension

(27) $$g_{\beta }\left(\left[\beta ,Z_{1}\right],\left[\beta ,Z_{2}\right]\right)=\tilde{\Theta }\left(Z_{1},\left[\beta ,Z_{2}\right]\right)+\langle Q,\left[Z_{1},\left[\beta ,Z_{2}\right]\right]\rangle$$

The additional Souriau term $\tilde{\Theta }\left(Z_{1},\left[\beta ,Z_{2}\right]\right)$ is generated by the non-equivariance of the coadjoint operator via the symplectic cocycle. The tensor $\tilde{\Theta }$ used to define this extended Fisher metric is defined by the moment map $J\left(X\right)$, map from M (homogeneous symplectic manifold) to $g^{*}$ the dual space of the Lie algebra, given by the following:

(28) $$\tilde{\Theta }\left(X,Y\right)=J_{\left[X,Y\right]}-\left\{J_{X},J_{Y}\right\}$$

(29) $$\mathrm{with}\;J(x):M\to g^{*}\;\mathrm{such}\;\mathrm{that}\;J_{X}(x)=\langle J(x),X\rangle ,\;X\in g$$

This tensor $\tilde{\Theta }$ is also defined in the tangent space of the cocycle $\theta \left(g\right)\in g^{*}$ (this cocycle appears due to the non-equivariance of the coadjoint operator $Ad_{g}^{*}$, action of the group on the dual space of the Lie algebra, which is modified with a cocycle).

We talk of affine action as illustrated in Figure 9:

(30) $$Q\left(Ad_{g}\beta \right)=Ad_{g}^{*}\left(Q\right)+\theta \left(g\right)$$

In the following we will use the following notation:

(31) $$Ad_{g}^{*}={\left(Ad_{g^{-1}}\right)}^{*}\mathrm{with}\;\langle Ad_{g}^{*}F,Y\rangle =\langle F,Ad_{g^{-1}}Y\rangle ,\forall g\in G,Y\in g,F\in g^{*}$$

such as Koszul and Souriau use it. $\theta \left(g\right)\in g^{*}$ is called a Souriau cocycle, and it is a measure of the lack of equivariance.

(32) $$\begin{array}{c} \tilde{\Theta }\left(X,Y\right):g\times g\to R\;\;\;\;\;\mathrm{with}\;\Theta (X)=T_{e}\theta \left(X(e)\right)\\ \;X,Y\mapsto \langle \Theta (X),Y\rangle \end{array}$$

We can then deduce that the tensor could also be written (with cocycle relation) as follows:

(33) $$\tilde{\Theta }\left(X,Y\right)=J_{\left[X,Y\right]}-\left\{J_{X},J_{Y}\right\}=-\langle d\theta \left(X\right),Y\rangle \;,\;X,Y\in g$$

(34) $$\tilde{\Theta }(\left[X,Y\right],Z)+\tilde{\Theta }(\left[Y,Z\right],X)+\tilde{\Theta }(\left[Z,X\right],Y)=0\;,\;X,Y,Z\in g$$

**Figure 10:**
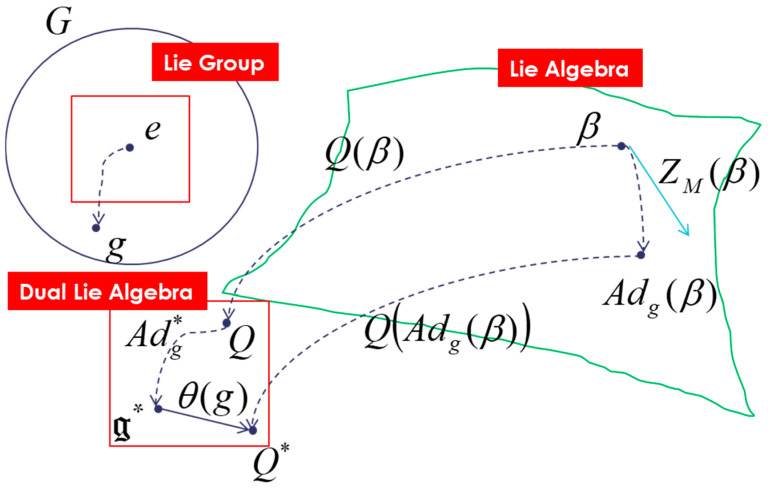
We take a value *g* in the Lie group and consider its action on the Lie algebra *β* (given by green contour) and on the dual of Lie algebra *Q* (given by red contour). The difference between the two actions provides the default of equivariance of the coadjoint operator, called Souriau cocycle *θ*.

We illustrate the previous equation in Figure 10.

When an element of the group *g* acts on the element β∈g of the Lie algebra, the operator is given by the adjoint operator $Ad_{g}$. With respect to the group action $Ad_{g}(\beta )$, entropy $S\left(Q\right)$ and Fisher’s metric $I\left(\beta \right)$ are invariant:

(35) $$\beta \in g\to Ad_{g}(\beta )\Rightarrow \left\{\begin{array}{l} S\left[Q\left(Ad_{g}(\beta )\right)\right]=S\left(Q\right)\\ I\left[Ad_{g}(\beta )\right]=I\left(\beta \right) \end{array}\right.$$

Souriau completed his “geometric theory of heat” by introducing a two-form into the Lie algebra, that is, a metric Riemann tensor in the values of the adjoint orbit of β, $\left[\beta ,Z\right]$ with Z an element of Lie algebra. This metric is given for $\left(\beta ,Q\right)$ as follows:

(36) $$g_{\beta }\left(\left[\beta ,Z_{1}\right],\left[\beta ,Z_{2}\right]\right)=\langle \Theta \left(Z_{1}\right),\left[\beta ,Z_{2}\right]\rangle +\langle Q,\left[Z_{1},\left[\beta ,Z_{2}\right]\right]\rangle$$

where Θ is a cocycle of the Lie algebra, defined by $\Theta =T_{e}\theta$ with θ a cocycle of the Lie group defined by $\theta (M)=Q\left(Ad_{M}(\beta )\right)-Ad_{M}^{*}Q$. We observe that the Riemannian Souriau metric, introduced with the symplectic cocycle, is a generalization of the Fisher metric, which we call the Souriau–Fisher metric, which retains the property of being defined as the Hessian of the logarithm of the partition function:

(37) $$g_{\beta }=-\frac{\partial^{2}\Phi }{\partial \beta^{2}}=\frac{\partial^{2}\log\psi_{\Omega }}{\partial \beta^{2}}$$

as in classical information geometry. We will establish the equality of two terms, between the definition of Souriau based on the cocycle of the Lie group Θ and parameterized by the “geometric heat” *Q* (element of the dual space of the Lie algebra) and the “geometric temperature” *β* (element of the Lie algebra) and the Hessian of the characteristic function $\Phi \left(\beta \right)=-\log\psi_{\Omega }(\beta )$ with respect to the variable *β*, as illustrated in Figure 10:

(38) $$g_{\beta }\left(\left[\beta ,Z_{1}\right],\left[\beta ,Z_{2}\right]\right)=\langle \Theta \left(Z_{1}\right),\left[\beta ,Z_{2}\right]\rangle +\langle Q,\left[Z_{1},\left[\beta ,Z_{2}\right]\right]\rangle =\frac{\partial^{2}\log\psi_{\Omega }}{\partial \beta^{2}}$$

**Figure 11:**
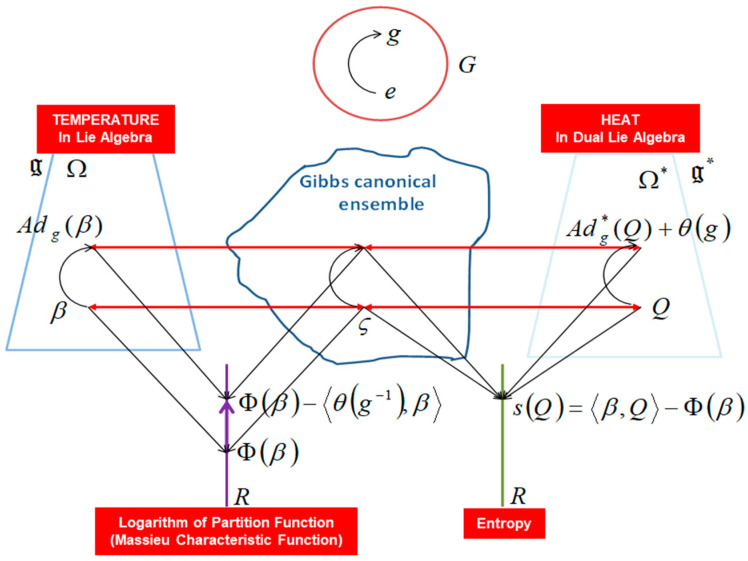
Relations of Souriau’s Lie group thermodynamics with *β* the geometric Planck temperature (element of the Lie algebra) and *Q* the geometric heat (element of the dual of the Lie algebra) and the action of the Lie group on entropy *S* (that is invariant) and Massieu characteristic function Φ.

An illustration of Souriau Lie group thermodynamics is given in Figure 11.

To consider entropy invariance, we must use the following property:

(39) $$Q\left(Ad_{g}\beta \right)=Ad_{g}^{*}Q\left(\beta \right)+\theta (g)=g.Q\left(\beta \right)\;,\;\beta \in \Omega ,g\in G$$

For β∈Ω, either $g_{\beta }$ the Hessian form on $T_{\beta }\Omega \equiv g$ with the potential $\Phi (\beta )=-\log\Psi_{\Omega }(\beta )$. For X,Y∈g, we define as follows:

(40) $$g_{\beta }\left(X,Y\right)=-\frac{\partial^{2}\Phi }{\partial \beta^{2}}\left(X,Y\right)={\left(\frac{\partial^{2}}{\partial s\partial t}\right)}_{s=t=0}\log\Psi_{\Omega }\left(\beta +sX+tY\right)$$

The positive definitive character is given by the Cauchy–Schwarz inequality:

(41) $$\begin{array}{l} g_{\beta }\left(X,Y\right)=\frac{1}{\Psi_{\Omega }{\left(\beta \right)}^{2}}\left\{\begin{array}{l} \int_{M}e^{-\langle U\left(\xi \right),\beta \rangle }d\lambda \left(\xi \right).\int_{M}{\langle U\left(\xi \right),X\rangle }^{2}e^{-\langle U\left(\xi \right),\beta \rangle }d\lambda \left(\xi \right)\\ -{\left(\int_{M}\langle U\left(\xi \right),X\rangle e^{-\langle U\left(\xi \right),\beta \rangle }d\lambda \left(\xi \right)\right)}^{2} \end{array}\right\}\\ =\frac{1}{\Psi_{\Omega }{\left(\beta \right)}^{2}}\left\{\begin{array}{l} {\int_{M}\left(e^{-\langle U\left(\xi \right),\beta \rangle /2}\right)}^{2}d\lambda \left(\xi \right).{\int_{M}\left(\langle U\left(\xi \right),X\rangle e^{-\langle U\left(\xi \right),\beta \rangle /2}\right)}^{2}d\lambda \left(\xi \right)\\ -{\left(\int_{M}e^{-\langle U\left(\xi \right),\beta \rangle /2}.\langle U\left(\xi \right),X\rangle e^{-\langle U\left(\xi \right),\beta \rangle /2}d\lambda \left(\xi \right)\right)}^{2} \end{array}\right\}\geq 0 \end{array}$$

We observe that $g_{\beta }\left(X,X\right)=0$ if and only if $\langle U\left(\xi \right),X\rangle$ is independent of ξ∈M, which means that the set $\left\{U\left(\xi \right);\xi \in M\right\}$ is contained in an affine hyperplane in $g^{*}$ perpendicular to the vector X∈g. We have seen that $g_{\beta }=-\frac{\partial^{2}\Phi }{\partial \beta^{2}}$, which is a generalization of the classic Fisher metric from information geometry, and will give the relation the Riemannian metric introduced by Souriau: $g_{\beta }\left(X,Y\right)=\langle -\frac{\partial Q}{\partial \beta }(X),Y\rangle \;\mathrm{for}\;X,Y\in g$

(42) $$g_{\beta }\left(X,Y\right)=\langle -\frac{\partial Q}{\partial \beta }(X),Y\rangle \;\mathrm{for}\;X,Y\in g$$

We have for everything β∈Ω,g∈G and Y∈g:

(43) $$\langle Q\left(Ad_{g}\beta \right),Y\rangle =\langle Q(\beta ),Ad_{g^{-1}}Y\rangle +\langle \theta (g),Y\rangle$$

Let us derive the above expression with respect to *g*. Namely, we substitute $g=\exp\left(tZ_{1}\right),t\in R$ and differentiate at *t* = 0. Then, the left side of the equation becomes

(44) $${\left(\frac{d}{dt}\right)}_{t=0}\langle Q\left(\beta +t\left[Z_{1},\beta \right]+o\left(t^{2}\right)\right),Y\rangle =\langle \frac{\partial Q}{\partial \beta }\left(\left[Z_{1},\beta \right]\right),Y\rangle$$

and the right-hand side of the other equation is calculated as follows:

(45) $$\begin{array}{l} {\left(\frac{d}{dt}\right)}_{t=0}\langle Q\left(\beta \right),Y-t\left[Z_{1},Y\right]+o\left(t^{2}\right)\rangle +\langle \theta \left(I+tZ_{1}+o\left(t^{2}\right)\right),Y\rangle \\ =-\langle Q(\beta ),\left[Z_{1},Y\right]\rangle +\langle d\theta \left(Z_{1}\right),Y\rangle \end{array}$$

(46) $$\mathrm{and}\;\mathrm{so},\langle \frac{\partial Q}{\partial \beta }\left(\left[Z_{1},\beta \right]\right),Y\rangle =\langle d\theta \left(Z_{1}\right),Y\rangle -\langle Q(\beta ),\left[Z_{1},Y\right]\rangle$$

Next, we substitute $Y=-\left[\beta ,Z_{2}\right]$ for the expression above:

(47) $$\begin{array}{l} g_{\beta }\left(\left[\beta ,Z_{1}\right],\left[\beta ,Z_{2}\right]\right)=\langle -\frac{\partial Q}{\partial \beta }\left(\left[Z_{1},\beta \right]\right),\left[\beta ,Z_{2}\right]\rangle \\ g_{\beta }\left(\left[\beta ,Z_{1}\right],\left[\beta ,Z_{2}\right]\right)=-\langle d\theta (Z_{1}),\left[\beta ,Z_{2}\right]\rangle +\langle Q(\beta ),\left[Z_{1},\left[\beta ,Z_{2}\right]\right]\rangle \end{array}$$

We then define the symplectic two-cocycle and the tensor:

(48) $$\begin{array}{l} \Theta \left(Z_{1}\right)=-d\theta \left(Z_{1}\right)\\ \tilde{\Theta }\left(Z_{1},Z_{2}\right)=\langle \Theta \left(Z_{1}\right),Z_{2}\rangle =\left\{J_{Z_{1}},J_{Z_{2}}\right\}-J_{\left[Z_{1},Z_{2}\right]} \end{array}$$

Considering ${\tilde{\Theta }}_{\beta }\left(Z_{1},Z_{2}\right)=\langle Q\left(\beta \right),\left[Z_{1},Z_{2}\right]\rangle +\tilde{\Theta }\left(Z_{1},Z_{2}\right)$, it is an extension of the KKS (Kirillov–Kostant–Souriau) two-form in the case of non-zero cohomology. Introduced by Souriau, we can define this metric extension of Fisher with the two-form of Souriau:

(49) $$g_{\beta }\left(\left[\beta ,Z_{1}\right],\left[\beta ,Z_{2}\right]\right)={\tilde{\Theta }}_{\beta }\left(Z_{1},\left[\beta ,Z_{2}\right]\right)$$

As the entropy is defined by the Legendre transform of the characteristic function, a dual metric of the Fisher metric is also given by the Hessian of the “geometric entropy” $S\left(Q\right)$ with respect to the dual variable given by *Q*:

(50) $$\frac{\partial^{2}S\left(Q\right)}{\partial Q^{2}}$$

Fisher’s metric was considered by Souriau as a generalization of “heat capacity”. Souriau called it “geometric capacity”:

(51) $$I(\beta )=-\frac{\partial^{2}\Phi (\beta )}{\partial \beta^{2}}=-\frac{\partial Q}{\partial \beta }$$

In his 1974 article, Jean-Marie Souriau wrote the following:

(52) $$\langle Q,\left[\beta ,Z\right]\rangle +\tilde{\Theta }\left(\beta ,Z\right)=0$$

To prove this equation, we need to consider the parameterized curve $t\mapsto Ad_{\exp\left(tZ\right)}\beta \;\mathrm{with}\;Z\in g\;\mathrm{et}\;t\in R$. The parameterized curve $Ad_{\exp\left(tZ\right)}\beta$ passes, for t=0, through the point β, since $Ad_{\exp\left(0\right)}$ is the identical map of the Lie algebra g. This curve is in the adjoint orbit of β. Thus, by taking its derivative with respect to t, then for t=0, we obtain a tangent vector in β to the deputy orbit of this point. When Z takes all possible values in g, the vectors thus obtained generate the entire tangent vector space β at the orbit of this point:

(53) $${\left.\frac{d\Phi \left(Ad_{\exp\left(tZ\right)}\beta \right)}{dt}\right|}_{t=0}=\langle \frac{d\Phi }{d\beta },\left({\left.\frac{d\left(Ad_{\exp\left(tZ\right)}\beta \right)}{dt}\right|}_{t=0}\right)\rangle =\langle Q,ad_{Z}\beta \rangle =\langle Q,\left[Z,\beta \right]\rangle$$

As $\Phi \left(Ad_{g}\beta \right)=\Phi \left(\beta \right)-\langle \theta \left(g^{-1}\right),\beta \rangle$, with $g=\exp\left(tZ\right)$, we have the following:

(54) $$\Phi \left(Ad_{\exp\left(tZ\right)}\beta \right)=\Phi \left(\beta \right)-\langle \theta \left(\exp\left(-tZ\right)\right),\beta \rangle$$

Thus, by derivation with respect to t at t=0, we finally find the equation given by Souriau:

(55) $${\left.\frac{d\Phi \left(Ad_{\exp\left(tZ\right)}\beta \right)}{dt}\right|}_{t=0}=\langle Q,\left[Z,\beta \right]\rangle =-\langle d\theta (-Z),\beta \rangle \;\mathrm{with}\;\tilde{\Theta }\left(X,Y\right)=-\langle d\theta (X),Y\rangle$$

### 2.5. Hidden Geometric Definition of Entropy as Casimir Function in Souriau’s Equation

We propose to characterize this invariance more explicitly, by characterizing entropy as an invariant Casimir function in coadjoint representation. From the last Souriau equation, if we use the identities $\beta =\frac{\partial S}{\partial Q}$, $ad_{\beta }Z=\left[\beta ,Z\right]$ and $\tilde{\Theta }\left(\beta ,Z\right)=\langle \Theta \left(\beta \right),Z\rangle$, then we can deduce the following:

(56) $$\langle ad_{\frac{\partial S}{\partial Q}}^{*}Q+\Theta \left(\frac{\partial S}{\partial Q}\right),Z\rangle =0,\forall Z$$

Then, the entropy $S\left(Q\right)$ must verify

(57) $$ad_{\frac{\partial S}{\partial Q}}^{*}Q+\Theta \left(\frac{\partial S}{\partial Q}\right)=0$$

that characterizes an invariant Casimir function in the case of non-zero cohomology, which we propose to write with Poisson brackets, where

(58) $${\left\{S,H\right\}}_{\tilde{\Theta }}\left(Q\right)=\langle Q,\left[\frac{\partial S}{\partial Q},\frac{\partial H}{\partial Q}\right]\rangle +\tilde{\Theta }\left(\frac{\partial S}{\partial Q},\frac{\partial H}{\partial Q}\right)=0,\;\forall H:g^{*}\to R,Q\in g^{*}$$

This definition of entropy is new and purely geometric. Typically, Shannon’s axiomatic definition of entropy is used. The geometric definition allows us to generalize and construct entropy starting from the symmetry group acting on the system. To construct entropy, it is sufficient to consider the coadjoint orbits generated by the Lie group on the dual of the Lie algebra via the moment map. The coadjoint operator then generates a symplectic foliation corresponding to the level curves of entropy.

We find the extended Casimir equation in the case of non-zero cohomology verified by entropy:

(59) $$ad_{\frac{\partial S}{\partial Q}}^{*}Q+\Theta \left(\frac{\partial S}{\partial Q}\right)=0$$

Then, the generalized Casimir condition ${\left\{S,H\right\}}_{\tilde{\Theta }}\left(Q\right)=0$. This previous Lie–Poisson equation is equivalent to the **modified Lie–Poisson variational principle**:

(60) $$\delta \int_{0}^{\tau }\left(\langle Q(t),\frac{\partial H}{\partial Q}(t)\rangle -H\left(Q(t)\right)\right)dt\;=0\underset{\begin{array}{l} Int.\\ by\\ parts \end{array}}{=}\int_{0}^{\tau }\langle -\frac{dQ}{dt}+ad_{\frac{dH}{dQ}}^{*}Q+\Theta \left(\frac{\partial H}{\partial Q}\right),\eta \rangle dt+{\left.\langle Q,\eta \rangle \right|}_{0}^{\tau }=0$$

From this Lie–Poisson equation, we can introduce a ***geometric Fourier heat equation***:

(61) $$\frac{\partial Q}{\partial t}=\frac{\partial Q}{\partial \beta }.\frac{\partial \beta }{\partial t}=ad_{\frac{\partial H}{\partial Q}}^{*}Q+\Theta \left(\frac{\partial H}{\partial Q}\right)$$

(62) $$\begin{array}{l} \mathrm{with}\;\frac{\partial Q}{\partial \beta }{\;\mathrm{given}\;\mathrm{by}\;g}_{\beta }\left(X,Y\right)=\langle -\frac{\partial Q}{\partial \beta }(X),Y\rangle \forall X,Y\in g\\ g_{\beta }\left(X,Y\right)={\tilde{\Theta }}_{\beta }\left(X,Y\right)=\langle Q\left(\beta \right),\left[X,Y\right]\rangle +\tilde{\Theta }\left(X,Y\right) \end{array}$$

The link with the second principle of thermodynamics will be deduced from the positivity of the Souriau–Fisher metric:

(63) $$\begin{array}{l} \frac{dS}{dt}=\langle Q,\frac{d\beta }{dt}\rangle +\langle ad_{\frac{\partial H}{\partial Q}}^{*}Q+\Theta \left(\frac{\partial H}{\partial Q}\right),\beta \rangle -\frac{d\Phi }{dt}\\ \frac{dS}{dt}=\langle Q,\frac{d\beta }{dt}\rangle +\langle Q,\left[\frac{\partial H}{\partial Q},\beta \right]\rangle +\tilde{\Theta }\left(\frac{\partial H}{\partial Q},\beta \right)-\frac{d\Phi }{dt}={\tilde{\Theta }}_{\beta }\left(\frac{\partial H}{\partial Q},\beta \right)\geq 0\\ \mathrm{If}\;H=S\underset{\frac{\partial S}{\partial Q}=\beta }{\Rightarrow }\frac{dS}{dt}={\tilde{\Theta }}_{\beta }\left(\beta ,\beta \right)=0\;\mathrm{because}\;\beta \in Ker{\tilde{\Theta }}_{\beta } \end{array}$$

The two equations characterizing entropy as an invariant Casimir function are linked by the following:

(64) $$\begin{array}{l} {\left\{S,H\right\}}_{\tilde{\Theta }}\left(Q\right)=\langle Q,\left[\frac{\partial S}{\partial Q},\frac{\partial H}{\partial Q}\right]\rangle +\langle \Theta \left(\frac{\partial S}{\partial Q}\right),\frac{\partial H}{\partial Q}\rangle =0\\ {\left\{S,H\right\}}_{\tilde{\Theta }}\left(Q\right)=\langle Q,ad_{\frac{\partial S}{\partial Q}}\frac{\partial H}{\partial Q}\rangle +\langle \Theta \left(\frac{\partial S}{\partial Q}\right),\frac{\partial H}{\partial Q}\rangle =\langle ad_{\frac{\partial S}{\partial Q}}^{*}Q,\frac{\partial H}{\partial Q}\rangle +\langle \Theta \left(\frac{\partial S}{\partial Q}\right),\frac{\partial H}{\partial Q}\rangle =0\\ \forall H,{\left\{S,H\right\}}_{\tilde{\Theta }}\left(Q\right)=\langle ad_{\frac{\partial S}{\partial Q}}^{*}Q+\Theta \left(\frac{\partial S}{\partial Q}\right),\frac{\partial H}{\partial Q}\rangle =0\Rightarrow ad_{\frac{\partial S}{\partial Q}}^{*}Q+\Theta \left(\frac{\partial S}{\partial Q}\right)=0 \end{array}$$

This equation was observed by Souriau, where he wrote that β is a kernel of ${\tilde{\Theta }}_{\beta }$, that is to say

(65) $$\beta \in Ker{\tilde{\Theta }}_{\beta }\Rightarrow \langle Q,\left[\beta ,Z\right]\rangle +\tilde{\Theta }\left(\beta ,Z\right)=0$$

which we can develop to find the Casimir equation:

(66) $$\begin{array}{l} \Rightarrow \langle Q,ad_{\beta }Z\rangle +\tilde{\Theta }\left(\beta ,Z\right)=0\Rightarrow \langle ad_{\beta }^{*}Q,Z\rangle +\tilde{\Theta }\left(\beta ,Z\right)=0\\ \beta =\frac{\partial S}{\partial Q}\Rightarrow \langle ad_{\frac{\partial S}{\partial Q}}^{*}Q,Z\rangle +\tilde{\Theta }\left(\frac{\partial S}{\partial Q},Z\right)=\langle ad_{\frac{\partial S}{\partial Q}}^{*}Q+\Theta \left(\frac{\partial S}{\partial Q}\right),Z\rangle =0,\forall Z\\ \Rightarrow ad_{\frac{\partial S}{\partial Q}}^{*}Q+\Theta \left(\frac{\partial S}{\partial Q}\right)=0 \end{array}$$

$H^{0}\left[\Omega \right]=Casim\left(M\right)$ is the set of Casimir functions on M, linked to Rham cohomology and Poisson cohomology introduced by J.L. Koszul. In the article “Quantum? So it’s geometric,” Souriau writes the Quinta Essentia (Quinte Essence) of his model:

[…] Let us first place ourselves within the framework of classical mechanics. Let us study an isolated, non-dissipative mechanical system—we will briefly call it a “thing”. The set of movements of this “thing” is a symplectic manifold. For what ? It is enough to refer to Lagrange’s Analytical Mechanics [75]; the space of movements is treated as a differentiable manifold; the covariant and contravariant coordinates of the symplectic form are written there (these are the “parentheses” and “brackets” of Lagrange). Let’s now talk about 20th century geometry. Let G be a diffeological group (for example a Lie group); μ a moment of G (a moment, it is a left-invariant 1-form on G); then the action of the group on μ canonically generates a symplectic space (these groups could have an infinite dimension). Epistemological presumption: behind each “thing” is hidden a group G (its “source”), and the movements of the “thing” are simply moments of G (mnemonic Latin doublet: momentum-movimentum). The isolation of the “thing” then indicates that the Poincaré group (respectively Galileo-Bargmann) is inserted in G; this is the origin of the conserved relativistic (respectively classical) quantities associated with a movement x: they simply constitute the moment induced on the spatio-temporal group by the moment-movement x. …There is a theorem that dates back to the 20th century. If we take a coadjoint orbit of a Lie group, it has a symplectic structure. Here is an algorithm for producing symplectic manifolds: take coadjoint orbits of a group. So this suggests that behind this Lagrange symplectic structure, there was a hidden group. Let’s take the classic movement of a moment of the group, then this group is very “big” to have the whole solar system. But in this group is included the Galileo group, and every moment of a group generates moments of a subgroup. We will thus find the moments of the Galileo group, and if we want relativistic mechanics, it will be that of the Poincaré group. In fact with the Galileo group, there is a small problem, it is not the moments of the Galileo group that we use, it is the moments of a central extension of the Galileo group, which is called the Bargmann group, and which has dimension 11. It is because of this extension that there is this famous arbitrary constant appearing in energy. On the other hand, when we do special relativity, we take the Poincaré group and there are no more problems because among the moments there is the mass and the energy is mc2. So the group of dimension 11 is an artifact which disappears when we do special relativity.

(Souriau [18], video)

**Figure 12:**
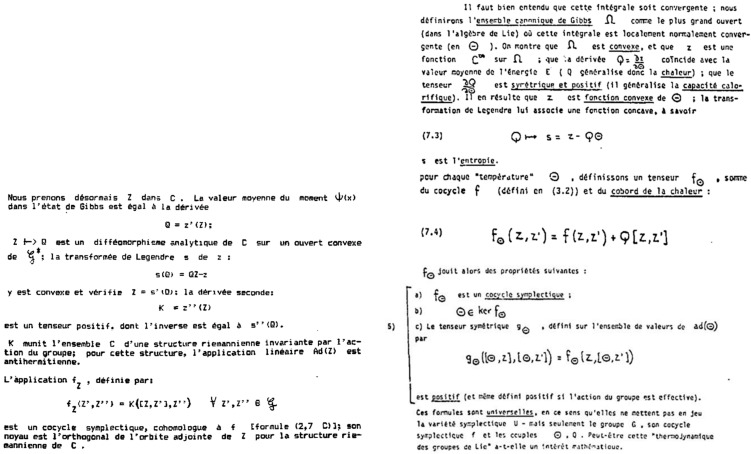
Lie group thermodynamics equations in Souriau’s papers. Source (Souriau [11]).

This model of Souriau in his manuscript is given in Figure 12.

To illustrate his Lie group thermodynamics, Souriau will consider the Galileo group, which is composed of the following transformations (rotation, *R*; boost, *u*; spatial translation, *w*; and time translation, *e*):

(67) $$\left[\begin{array}{c} {\vec{x}}^{\prime }\\ t^{\prime }\\ 1 \end{array}\right]=\left[\begin{array}{ccc} R & \vec{u} & \vec{w}\\ 0 & 1 & e\\ 0 & 0 & 1 \end{array}\right]\left[\begin{array}{c} \vec{x}\\ t\\ 1 \end{array}\right]\;\mathrm{or}\;\left\{\begin{array}{l} {\vec{x}}^{\prime }=R\vec{x}+\vec{u}t+\vec{w}\\ t^{\prime }=t+e \end{array}\right.$$

Souriau noticed that in the global case, there is no Gibbs equilibrium (Gibbs [33,96,97,98,99]); he therefore considers Gibbs states (Kozlov [100]) for Hamiltonian actions of subgroups with one parameter of the Galileo group. In particular, he obtains the following theorem:

**Souriau’s theorem**: The action of the complete Galilean group on the space of movements of an isolated mechanical system is not linked to any state of Gibbs equilibrium (the open subset of the Lie algebra, associated with this state of Gibbs, is empty).

The one-parameter subgroup of the Galileo group generated by *β* element of the Lie algebra is given by the following matrices:

(68) $$\begin{array}{c} \exp(\tau \beta )=\left(\begin{array}{ccc} A(\tau ) & \vec{b}(\tau ) & \vec{d}(\tau )\\ 0 & 1 & \tau \varepsilon \\ 0 & 0 & 1 \end{array}\right)\\ \mathrm{with}\;\left\{\begin{array}{l} A(\tau )=\exp\left(\tau j(\vec{\omega })\right)\;\mathrm{and}\;\vec{b}(\tau )=\left(\sum_{i=1}^{\infty }\frac{\tau^{i}}{i!}{\left(j(\vec{\omega })\right)}^{i-1}\right)\vec{\alpha }\\ \vec{d}(\tau )=\left(\sum_{i=1}^{\infty }\frac{\tau^{i}}{i!}{\left(j(\vec{\omega })\right)}^{i-1}\right)\vec{\delta }+\varepsilon \left(\sum_{i=2}^{\infty }\frac{\tau^{i}}{i!}{\left(j(\vec{\omega })\right)}^{i-2}\right)\vec{\alpha } \end{array}\right.\;\mathrm{and}\;\beta =\left(\begin{array}{ccc} j\left(\vec{\omega }\right) & \vec{\alpha } & \vec{\delta }\\ 0 & 0 & \varepsilon \\ 0 & 0 & 0 \end{array}\right)\in g \end{array}$$

Souriau illustrated the Gibbs equilibrium for the centrifuge, for which the group which acts is that of rotation around the axis:

(69) $$\vec{\omega }=\omega {\vec{e}}_{z}\;,\;\vec{\alpha }=0\;\mathrm{and}\;\vec{\delta }=0\;\mathrm{with}\;\mathrm{rotation}\;\mathrm{speed}:\frac{\omega }{\varepsilon }$$

By posing the following expressions

(70) $$f_{i}\left({\vec{r}}_{i0}\right)=-\frac{\omega^{2}}{2\varepsilon^{2}}{\left\|{\vec{e}}_{z}\times {\vec{r}}_{i0}\right\|}^{2}\;\mathrm{with}\;\Delta =\left\|{\vec{e}}_{z}\times {\vec{r}}_{i0}\right\|\;\mathrm{distance}\;z\;\mathrm{axis}$$

Souriau finds with the equations of his model the Gibbs equilibrium of the centrifuge via the moment map *J*, as illustrated in Figure 12:

(71) $$\rho_{i}\left(\beta \right)=\frac{1}{P_{i}\left(\beta \right)}\exp\left(-\langle J_{i},\beta \rangle \right)=cst.\exp\left(-\frac{1}{2m_{i}\kappa T}{\left\|{\vec{p}}_{i0}\right\|}^{2}+\frac{m_{i}}{2\kappa T}{\left(\frac{\omega }{\varepsilon }\right)}^{2}\Delta^{2}\right)$$

the behavior of a gas made up of point particles of various masses in a centrifuge rotating at constant angular speed $\frac{\omega }{\varepsilon }$ (the heaviest particles concentrate further from the axis of rotation than the lighter ones). He specifies that the thermodynamics of the centrifuge can be used to make churned butter, enriched uranium, or ribo-acids.

Roger Balian (Balian [101]) explains as follows in his book:

[…] Angular momentum is transmitted to the gas when the molecules collide with the rotating walls, which changes the Maxwell distribution at each point, moving its origin. The walls act as a reservoir of angular momentum. Their movement is characterized by a certain angular speed, and the angular speeds of the fluid and the walls become equal at equilibrium, exactly like the equalization of temperature by energy exchanges.

(Balian [101])

We do have two (Planck) temperatures: the classic Planck temperature, ensuring the thermal balance of the centrifuge, and a second temperature, Lagrange hyper-parameter, which ensures the balance of the angular moments (the wall, reservoir of angular momentum transmits this angular momentum to the particles in contact with it which arrives at an equilibrium by viscosity).

**Figure 13:**
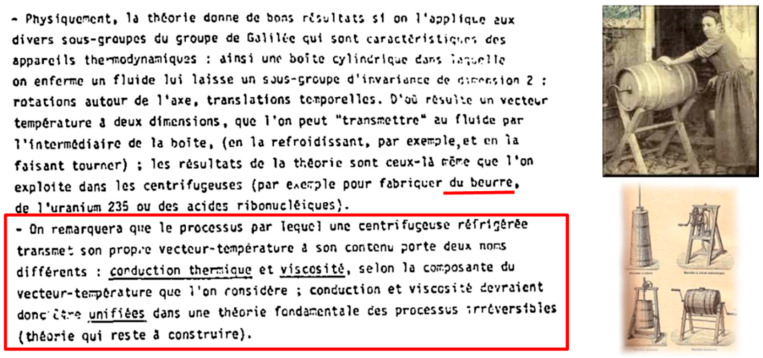
The thermodynamics of Souriau, that of the creamer of churned butter and the reference to dissipation (Fourier conduction and Navier viscosity). In red box Souriau write “It is noted that the process by which a refrigerated centrifuge transmits its own temperature vector to its contents has two different names: thermal conduction and viscosity, depending on the component of the temperature vector that is considered; conduction and viscosity should therefore be unified in a fundamental theory of irreversible processes (a theory that remains to be constructed)”. Source (Souriau [11]).

In Figure 13, Jean-Marie Souriau makes reference to this use-case and of the creamer of churned butter and the reference to dissipation (Fourier conduction and Navier viscosity).

## 3. Metriplectic Flow and Webs Model of Dissipative Thermodynamics

### 3.1. Theory of Foliation from Ehresmann and Reeb to Libermann

**Figure 14:**
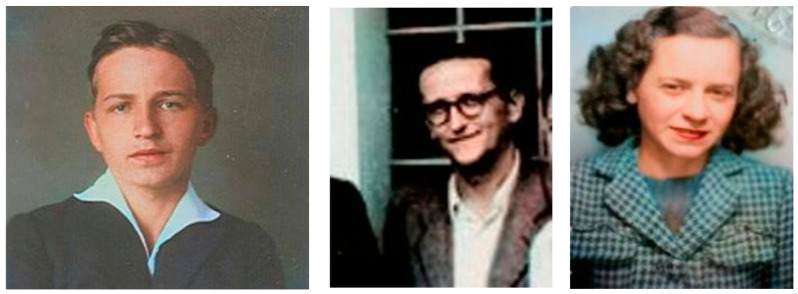
Charles Ehresmann, Georges Reeb, and Paulette Libermann. Source Gallica.

The theory of foliations is a qualitative theory, generalizing differential equations, initiated by Henri Poincaré and developed by Charles Ehresmann and Georges Reeb (Martinet & Reeb [102], Reeb [103,104,105,106]), with the contribution of A. Haefliger (Haefliger [107]), P. Molino (Molino [108,109], Condevaux Dazord & Molino [110]), B.L. Reinhart (Reinhart [111]). The specific foliations, called Riemannian, generated by metric functions were developed, for their part, by Ph. Tondeur. The notion of foliation in thermodynamics appeared in the 1900s in the seminal article by Constantin Carathéodory where the horizontal curves roughly correspond to adiabatic processes, carried out in the language of Carnot cycles. The couple properties of Poisson manifolds were also explored by C. Carathéodory in 1935, under the name “groups of polar functions”, where he observed that two families of differentiable functions formed by the prime integrals of F (a sub-completely integrable vector set of TM) and its orthogonal orthF, respectively, called “function groups”, are “polar” of each other. This seminal work by C. Carathéodory led to the concept of Poisson structure which was first defined and treated in depth for the first time by André (Basart & Lichnerowicz [112], Hamoui & Lichnerowicz [113]) and independently by Alexander Kirillov. André Haefliger observed that generally, for a field of planes of codimension one given by a Pfaff form ω, the integrability condition is equivalent to ω ∧ d ω = 0. In this case, there exists locally a non-zero function λ, called integration factor, and a function φ, called first integral, such that ω = λdφ. The level manifolds of φ are the integral manifolds. Carathéodory gave in 1909 a local geometric characterization of the complete integrability of a Pfaff form ω, namely, ω is completely integrable if and only if, for every neighborhood U of every point x, there exists a point of U which cannot be linked to x by a U-shaped curve tangent to the kernel of ω. He uses this characterization to express remarkably concisely and conceptually the second law of thermodynamics. Georges Reeb and Charles Ehresmann (in Figure 14), who founded the theory of foliations, organized numerous conferences on this theme with Paulette Libermann (PhD student of Charles Ehresmann).

**Figure 15:**
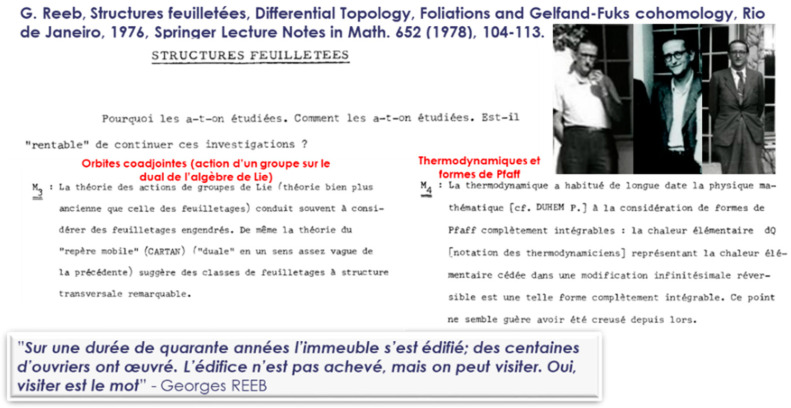
Georges Reeb’s reflections on the reasons which motivated the study of foliations. Source (Reeb [105]) and Gallica.

Symplectic geometry associated with analytical mechanics has developed considerably over the last decades, inspired by the work of Sophus Lie [114,115,116] and Elie Cartan, André Lichnerowicz, Georges Reeb, Jean-Marie Souriau, and François Gallissot, who were the initiators of this renaissance of analytical mechanics. Georges Reeb asked the following questions about foliage structures: “Why were they studied? How were they studied? Is it profitable to continue these investigations?” and the motivations proposed for the study of these foliations. Among the motivations, Reeb identified two key use cases, the action of Lie groups and the Pfaff integrable forms of thermodynamics, as illustrated in Figure 15:

[…] The theory of the action of Lie groups (a much older theory than that of foliations) often leads to considering the generated foliations. Likewise, the theory of the “moving frame” (Cartan) (“dual” in a rather vague sense of the previous one) suggests classes of foliations with a remarkable transverse structure.

(Reeb [105])

[…] Thermodynamics has long accustomed mathematical physics [cf. Duhem P.] to the consideration of completely integrable Pfaff forms: the elementary heat dQ [notation of thermodynamicists] representing the elementary heat given up in an infinitesimal reversible modification is such a completely integrable form. This point hardly seems to have been explored since then.

(Reeb [105])

The first subject on Cartan’s moving frame was developed by Edmond Fédida, doctoral student of Georges Reeb, and the second subject on the Pfaff forms of thermodynamics by Jean-Marie Souriau.

### 3.2. Transverse Symplectic Foliation Model of Dissipative Thermodynamics and the Metriplectic Flow

We introduce a web structure model from information geometry and heat theory based on the thermodynamics of Lie groups of Jean-Marie Souriau to describe the transverse Poisson structure of the metriplectic flow for dissipative phenomena. This model gives a Lie algebra cohomological characterization of entropy, as an invariant Casimir function in coadjoint representation. The dual space of the Lie algebra unfolds in coadjoint orbits identified with the level sets of entropy. In the context of thermodynamics, we associate a symplectic bi-foliation structure to describe the non-dissipative dynamics on the symplectic leaves (on level sets of entropy, the entropy being seen as an invariant Casimir function on each leaf) and the transverse dissipative dynamics, given by the transverse Poisson structure (production of entropy passing from leaf to leaf). The orbits of a Hamiltonian action and the level sets of the moment map are polar with respect to each other (Albert [117]). Souriau’s model can be interpreted by the foliations studied by Miss Paulette Libermann and the notion of Γ-structure of Haefliger, which is the maximum extension of the notion of moment in the sense of Souriau, as introduced by P. Molino, M. Condevaux, and P. Dazord in the articles of the “Séminaire Sud-Rhodanien de Géométrie”. Paulette Libermann proved that a Legendre foliation (Jayne [118,119]) on a contact manifold is complete if and only if the pseudo-orthogonal distribution is completely integrable and that the contact form is locally equivalent to the Poincaré–Cartan integral invariant. Paulette Libermann demonstrated a classic theorem relating to co-isotropic foliations, which notably provides a proof of Darboux’s theorem. Finally, we will refer to the work of Edmond Fédida on the theory of foliation structures in the language of fully integrable Pfaff systems associated with the Cartan moving frame.

The metriplectic bracket was first introduced in 1983 by A.N. Kaufman and P.J. Morrison. This formalism ensures both energy conservation and a non-decrease in entropy, and it reduces to the traditional formalism of Poisson brackets in the limit of the absence of dissipation. The axiomatization of this model was carried out in parallel by Grmela and Öttinger. There are three main types of dissipation: thermal diffusion with energy conservation and entropy production by heat transfer; viscosity, which extracts energy from the system (e.g., Navier–Stokes equation); and transport equations with collision operators. These types of dissipative systems that satisfy both the first and second principles of thermodynamics are included in metriplectic dynamics. A new bracket in the metriplectic formalism provides the evolution equation $\left\{\left\{.,.\right\}\right\}$:

(72) $$\frac{df}{dt}=\left\{\left\{f,F\right\}\right\}=\left\{f,F\right\}+\left(f,F\right)\;\mathrm{with}\;\mathrm{Hamiltonian}\;F=H+S$$

**Figure 16:**
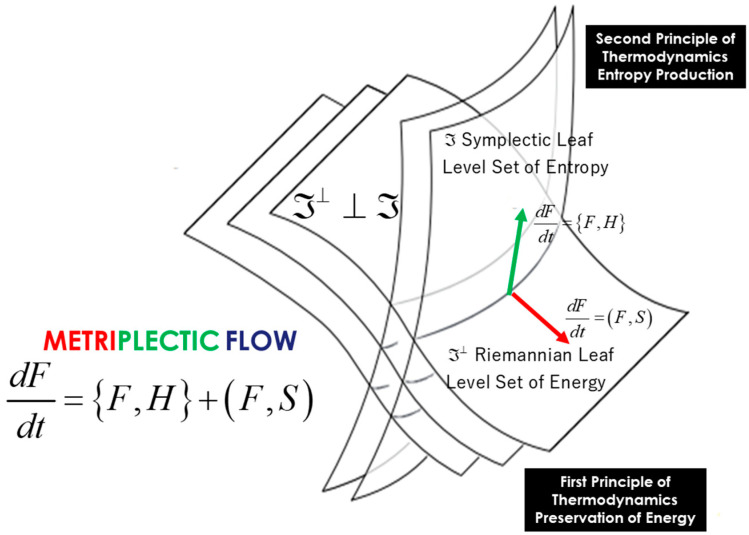
Metriplectic flow acts jointly on symplectic foliation (coadjoint orbits of moment map, level sets of entropy) and on transverse metric foliation (level sets of energy). {*F*, *H*} Poisson bracket describes the non-dissipative dynamics at constant entropy on symplectic leaves. (*F*, *S*) Metric bracket describes dissipative dynamics on Riemannian leaves at constant energy (first principle of thermodynamics) and with entropy production (second principle of thermodynamics).

The second bracket is a metric bracket checking the two constraints $\left(f,F\right)=\left(F,f\right)\;\mathrm{and}\;\left(f,f\right)\geq 0$ with the entropy *S* selected in the set of Casimir invariants of the non-canonical Poisson bracket. The metriplectic flow thus conforms to the first and second principles of thermodynamics:

First principle of thermodynamics: conservation of energy
  (73) $$\begin{array}{l} \frac{dH}{dt}=\left\{H,F\right\}+\left(H,F\right)=\left\{H,H\right\}+\left\{H,S\right\}+\left(H,H\right)+\left(H,S\right)=0\\ \mathrm{because}\;\left\{H,H\right\}=0\;\mathrm{by}\;\mathrm{symmetry},\;\left\{f,S\right\}=0,\forall f\;\mathrm{and}\;\left(H,f\right)=0,\forall f \end{array}$$
Second principle of thermodynamics: the production of entropy
  (74) $$\begin{array}{l} \frac{dS}{dt}=\left\{S,F\right\}+\left(S,F\right)=0+\left(S,H\right)+\left(S,S\right)=\left(S,S\right)\geq 0\\ \mathrm{with}\;\left\{S,f\right\}=0\forall f,\left(f,H\right)=0\forall f\;\mathrm{and}\;(,)\;\mathrm{positive}\;\mathrm{half}\text{-}\mathrm{definite} \end{array}$$
Finally, two compatible brackets, a Poisson bracket and a symmetric bracket, determine the geometry in metriplectic systems, as illustrated in Figure 16:
  (75) $$\frac{df}{dt}=\left\{\left\{f,F\right\}\right\}=\left\{f,H\right\}+\left(f,S\right)$$
The energy *H* is a Casimir invariant of the dissipative bracket, and the entropy *S* is a Casimir invariant of the Poisson bracket:
  (76) $$\left\{S,H\right\}=0\forall H\;\mathrm{and}\;\left(H,S\right)=0\forall S$$
  The symmetry requirement generalizes Onsager symmetry from irreversible linear thermodynamics to nonlinear problems; however, in the traditional metriplectic model, the possibility of Casimir symmetry is not taken into account. The bracket proposed by Kaufman is more general than the metriplectic bracket. The metriplectic equation linked to transverse symplectic foliations is illustrated in Figure 16.

The dissipative nature of quantum systems is both profound and insightful, particularly as revealed through the link between the Lindblad equation in quantum mechanics and the previous metriplectic formalism used in thermodynamics and classical dissipative systems. The dissipative equation introduced by Lindblad, derived from the Hamiltonian Liouville equation, operates on the quantum density matrix. The dissipative component of the Lindblad operator has been interpreted by Öttinger as the gradient of relative entropy and a manifestation of the maximum entropy principle. It has consequently been observed that the Lindblad equation constitutes a linear approximation of the metriplectic equation. As H.C. Öttinger has noted, after formulating a thermodynamically consistent nonlinear master equation, one may investigate the particular circumstances under which precise or approximate linear master equations can be obtained. H. Graber has pursued such an investigation and concluded that the resulting master equations are not of the conventional Lindblad form. The nonlinearity of the thermodynamic quantum master equation is arguably its most salient feature. This equation stands in contrast to the linear Liouville and Schrödinger equations, which describe reversible classical and quantum systems, as well as to the Fokker–Planck equations, which describe irreversible classical systems. The standard Lindblad-type master equations overlook this inherent nonlinearity.

Notably, solutions to thermodynamic master equations, like those of the linear Lindblad form, always remain within the physical domain—a subtle and significant issue for nonlinear formulations. Öttinger’s work demonstrates how nonlinearity can be properly addressed in the context of a two-level quantum system. Given that noncommutativity gives rise to quantum nonlinearity, Öttinger’s master equation cannot take the standard Lindblad form. The symmetric anticommutator offers the most natural linearization of the metriplectic equation. However, such linearizations compromise the thermodynamic structure and are therefore not advisable. The resulting linearized equation takes the Lindblad form, necessitating a redefinition of the Hamiltonian and the introduction of a Lindblad operator with both real and imaginary components. Thermodynamic considerations indicate that the quantum master equation ought to be strongly nonlinear. This thermodynamic structure allows for a rigorous formulation of a nonlinear quantum master equation. Öttinger’s linearized quantum master equation, in turn, enables the reconstruction of the standard Lindblad master equation. Nevertheless, the validity of this linear form is restricted by the underlying thermodynamic nonlinearity, which naturally leads to canonical equilibrium solutions. The Lindblad and metriplectic equations share structural similarities, as illustrated in Table 1.

We underline the local structure related to a completely integrable system given explicitly by action–angle coordinates, whose existence is induced by the Liouville–Arnold’s theorem. The Liouville–Arnold’s theorem assures the existence of action–angle coordinates adapted to a fibration, with respect to the existence of a sufficiently high number of specific integrals. If we consider action–angle coordinates (Nehorosev [120]), $\omega =dx^{i}\wedge d\theta_{i}$. We also consider the moment map $\mu :M\to \;g^{*}$ where $\left(x^{1},\ldots ,x^{n}\right)$ are coordinates on $g^{*}$ given by $x^{i}=\langle X_{i},.\rangle$ where $\left(X_{1},X_{2},\ldots ,X_{n}\right)$ is a base of vectors field of group action: $dx^{i}=-i_{X_{i}}\omega$. We can select angular coordinates such that $X_{i}=\frac{\partial }{\partial \theta_{i}}$. For symplectic coordinates and complex structure, we consider a complex structure J:

(77) $$Jdx^{i}=G^{ij}d\theta_{j}\;\mathrm{and}\;Jd\theta_{i}=-G_{ij}dx^{j}\;\mathrm{where}\;G_{ij}={\left(G^{ij}\right)}^{-1}$$

We can then deduce the metric as follows:

$$g=G_{ij}dx^{i}dx^{j}+G^{ij}d\theta_{i}d\theta_{j}\;\mathrm{where}\;\left(G_{ij}\right)\;\mathrm{is}\;\mathrm{symetric}\;\mathrm{positive}\;\mathrm{definite}$$

We can make appear a symplectic potential:

(78) $$dJd\theta_{i}=-\frac{\partial G_{ij}}{\partial x^{k}}dx^{k}\wedge dx^{j}\;\mathrm{of}\;\mathrm{type}\;(1,1)$$

(79) $$dJd\theta_{i}=0\Rightarrow \frac{\partial G_{ij}}{\partial x^{k}}=\frac{\partial G_{ik}}{\partial x^{j}},\exists u{\;\mathrm{convex},\;G}_{ij}=\frac{\partial^{2}u}{\partial x^{i}\partial x^{j}}=u_{ij}$$

We then recover the Guillemin metric:

(80) $$g=u_{ij}dx^{i}dx^{j}+u^{ij}d\theta_{i}d\theta_{j}\;\mathrm{where}\;\left(u^{ij}\right)={\left(u_{ij}\right)}^{-1}$$

Recently, new links have been established between information geometry, Toric Manifolds, and Delzant Polytopes (Delzant [121,122]), where the torification approach places information geometry at the very heart of information-theoretical principles.

**Figure 17:**
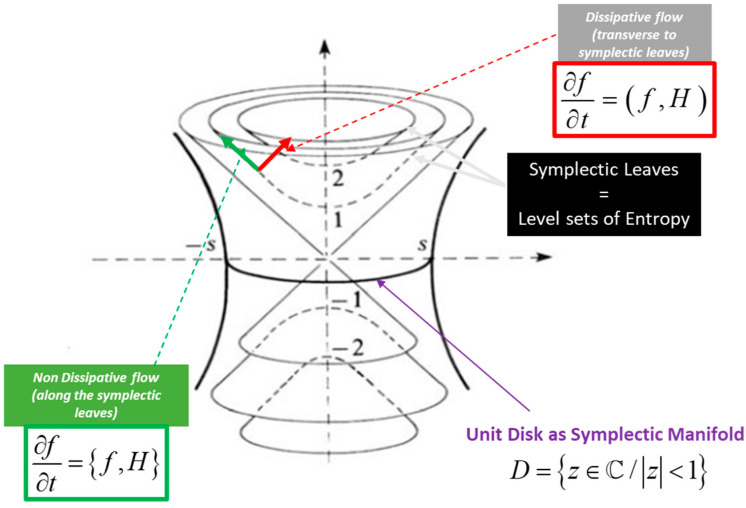
Metriplectic flow and symplectic foliation illustrated for the *SU*(1, 1) Lie group acting transitively on the Poincaré unit disk: upper sheets of the two-layer hyperboloid provide the symplectic foliation associated to the level set of entropy. {*f*, *H*} The Poisson bracket describes non-dissipative dynamics on symplectic leaves at constant entropy. (*f*, *H*) The metric bracket describes dissipative dynamics transversally to symplectic leaves.

To illustrate the metriplectic flow, we give, in the Figure 17, the illustration of the coadjoint orbits (generated via the moment map in the dual of the Lie algebra) for the Lie group *SU*(1, 1) acting transitively on the Poincaré complex unit disk. The symplectic foliation is given by the upper sheets of the two-layer hyperboloid. *J*(*z*) is the moment map which goes from z belonging to the unit disk on the hyperboloid (in fact, the three components of the moment map on the dual of the Lie algebra verify the equation of the hyperboloid). The Poisson bracket describes the non-dissipative dynamics of Souriau which remains on the sheet of the symplectic foliation (the entropy is constant on the sheet, layer of the hyperboloid, because the entropy is an invariant Casimir function on this sheet). We will introduce the Souriau moment map for *SU*(1, 1)/*U*(1) group that acts transitively on Poincaré unit disk, based on moment map. Considering the Lie group, first

(81) $$SU(1,1)=\left\{\begin{array}{l} \left(\begin{array}{cc} a & b\\ b^{*} & a^{*} \end{array}\right)=\left(\begin{array}{cc} 1 & ba^{*-1}\\ 0 & 1 \end{array}\right)\left(\begin{array}{cc} a^{*-1} & 0\\ 0 & a^{*} \end{array}\right)\left(\begin{array}{cc} 1 & 0\\ a^{*-1}b^{*} & 1 \end{array}\right)\\ /a,b\in C,\;{\left|a\right|}^{2}-{\left|b\right|}^{2}=1 \end{array}\right\}$$

and its Lie algebra given by elements

(82) $$su(1,1)=\left\{\left(\begin{array}{cc} ir & \eta \\ \eta^{*} & -ir \end{array}\right)/r\in R,\eta \in C\right\}$$

A basis for this Lie algebra su(1,1) is $\left(u_{1},u_{2},u_{3}\right)\in g$ with

(83) $$u_{1}=\frac{i}{2}\left(\begin{array}{cc} 1 & 0\\ 0 & -1 \end{array}\right),\;u_{2}=-\frac{1}{2}\left(\begin{array}{cc} 0 & 1\\ 1 & 0 \end{array}\right)\;\mathrm{and}\;u_{3}=\frac{1}{2}\left(\begin{array}{cc} 0 & -i\\ i & 0 \end{array}\right)$$

with $\left[u_{1},u_{3}\right]=-u_{2},\left[u_{1},u_{2}\right]=u_{3},\left[u_{2},u_{3}\right]=-u_{1}$. The Harish–Chandra embedding is given by $\varphi \left(gx_{0}\right)=\zeta =ba^{*-1}$. From ${\left|a\right|}^{2}-{\left|b\right|}^{2}=1$, one has $\left|\zeta \right|<1$. Conversely, for any $\left|\zeta \right|<1$, taking any a∈C such that $\left|a\right|={\left(1-{\left|a\right|}^{2}\right)}^{-1/2}$ and putting $b=\zeta a^{*}$, one obtains g∈G for which $\varphi \left(gx_{0}\right)=\zeta$. The domain D=φ(M) is the unit disc $D=\left\{\zeta \in C/\left|\zeta \right|<1\right\}$.

The compact subgroup is generated by $u_{1}$, while $u_{2}$ and $u_{3}$ generate a hyperbolic subgroup. The dual space of the Lie algebra is given by the following:

(84) $$su{\left(1,1\right)}^{*}=\left\{\left(\begin{array}{cc} z & x+iy\\ -x+iy & -z \end{array}\right)/x,y,z\in R\right\}$$

with the basis $\left(u_{1}^{*},u_{2}^{*},u_{3}^{*}\right)\in g^{*}$:

(85) $$u_{1}^{*}=\left(\begin{array}{cc} 1 & 0\\ 0 & -1 \end{array}\right),u_{2}^{*}=\left(\begin{array}{cc} 0 & i\\ i & 0 \end{array}\right)\;\mathrm{and}\;u_{3}^{*}=\left(\begin{array}{cc} 0 & 1\\ -1 & 0 \end{array}\right)$$

Let us consider $D=\left\{z\in C/\left|z\right|<1\right\}$ to be the open unit disk of Poincaré. For each ρ>0, the pair $\left(D,\omega_{\rho }\right)$ is a symplectic homogeneous manifold with $\omega_{\rho }=2i\rho \frac{dz\wedge dz^{*}}{{\left(1-{\left|z\right|}^{2}\right)}^{2}}$, where $\omega_{\rho }$ is invariant under the following action:

(86) $$\begin{array}{l} SU\left(1,1\right)\times D\to D\\ \left(g,z\right)\mapsto g.z=\frac{az+b}{b^{*}z+a^{*}} \end{array}$$

This action is transitive and is globally and strongly Hamiltonian. Its generators are the Hamiltonian vector fields associated to the following functions:

(87) $$\begin{array}{l} J_{1}(z,z^{*})=\rho \frac{1+{\left|z\right|}^{2}}{1-{\left|z\right|}^{2}},J_{2}(z,z^{*})=\frac{\rho }{i}\frac{z-z^{*}}{1-{\left|z\right|}^{2}}\\ \mathrm{and}\;J_{3}(z,z^{*})=-\rho \frac{z+z^{*}}{1-{\left|z\right|}^{2}} \end{array}$$

The associated moment map $J:D\to su^{*}(1,1)$ defined by $J(z).u_{i}=J_{i}(z,z^{*})$, maps D into a coadjoint orbit in $su^{*}(1,1)$. Then, we can write the moment map as a matrix element of $su^{*}(1,1)$:

(88) $$\begin{array}{l} J(z)=J_{1}\left(z,z^{*}\right)u_{1}^{*}+J_{2}\left(z,z^{*}\right)u_{2}^{*}+J_{3}\left(z,z^{*}\right)u_{3}^{*}=\\ J(z)=\rho \left(\begin{array}{cc} \frac{1+{\left|z\right|}^{2}}{1-{\left|z\right|}^{2}} & -2\frac{z^{*}}{1-{\left|z\right|}^{2}}\\ 2\frac{z}{1-{\left|z\right|}^{2}} & -\frac{1+{\left|z\right|}^{2}}{1-{\left|z\right|}^{2}} \end{array}\right)\in g^{*} \end{array}$$

The moment map J is a diffeomorphism of D onto one sheet of the two-sheeted hyperboloid in $su^{*}(1,1)$, determined by the following equation: $J_{1}^{2}-J_{2}^{2}-J_{3}^{2}=\rho^{2}\;,\;J_{1}\geq \rho \;\mathrm{with}$ $J_{1}u_{1}^{*}+J_{2}u_{2}^{*}+J_{3}u_{3}^{*}\in su^{*}(1,1)$. We note $O_{\rho }^{+}$ the coadjoint orbit $Ad_{SU(1,1)}^{*}$ of $SU\left(1,1\right)$, given by the upper sheet of the two-sheeted hyperboloid given by previous equation. The orbit method of Kostant–Kirillov–Souriau associates to each of these coadjoint orbits a representation of the discrete series of $SU\left(1,1\right)$, provided that ρ is a half integer greater or equal than 1 ($\rho =\frac{k}{2},k\in N\;\mathrm{and}\;\rho \geq 1$). When explicitly executing the Kostant–Kirillov construction, the representation Hilbert spaces $H_{\rho }$ are realized as closed reproducing kernel subspaces of $L^{2}\left(D,\omega_{\rho }\right)$. The Kostant–Kirillov–Souriau orbit method shows that to each coadjoint orbit of a connected Lie group is associated a unitary irreducible representation of G acting in a Hilbert space *H*.

Souriau has observed that action of the full Galilean group on the space of motions of an isolated mechanical system is not related to any equilibrium Gibbs state (the open subset of the Lie algebra, associated to this Gibbs state is empty). The main Souriau idea was to define the Gibbs states for one-parameter subgroups of the Galilean group. We will use the same approach in this case. We will consider action of the Lie group $SU\left(1,1\right)$ on the symplectic manifold (*M*, *ω*) (Poincaré unit disk) and its momentum map J such that the open subset $\Lambda_{\beta }=\left\{\beta \in g/\int_{D}e^{-\langle J(z),\beta \rangle }d\lambda (z)<+\infty \right\}\;$ is not empty. This condition is not always satisfied when (*M*, *ω*) is a cotangent bundle, but it is satisfied when it is a compact manifold. The idea of Souriau is to consider a one-parameter subgroup of $SU\left(1,1\right)$. To parametrize elements of $SU\left(1,1\right)$ is through its Lie algebra. In the neighborhood of the identity element, the elements of $g\in SU\left(1,1\right)$ can be written as the exponential of an element β of its Lie algebra:

(89) $$g=\exp\left(\varepsilon \beta \right)\;\mathrm{with}\;\beta \in g$$

The condition $g^{+}Mg=M\;\mathrm{for}\;M=\left(\begin{array}{cc} 1 & 0\\ 0 & -1 \end{array}\right)$ can be expanded for ε<<1 and is equivalent to $\beta^{+}M+M\beta =0$ which then implies $\beta =\left(\begin{array}{cc} ir & \eta \\ \eta^{*} & -ir \end{array}\right),r\in R,\eta \in C$. We can observe that r and $\eta =\eta_{R}+i\eta_{I}$ contain three degrees of freedom, as required. Also, because detg=1, we obtain Tr(β)=0. We exponentiate β with exponential map to obtain the following:

(90) $$g=\exp\left(\varepsilon \beta \right)=\sum_{k=0}^{\infty }\frac{{\left(\varepsilon \beta \right)}^{k}}{k!}=\left(\begin{array}{cc} a_{\varepsilon }(\beta ) & b_{\varepsilon }(\beta )\\ b_{\varepsilon }^{*}(\beta ) & a_{\varepsilon }^{*}(\beta ) \end{array}\right)$$

If we make the remark that we have the following relation $\beta^{2}=\left(\begin{array}{cc} ir & \eta \\ \eta^{*} & -ir \end{array}\right)\left(\begin{array}{cc} ir & \eta \\ \eta^{*} & -ir \end{array}\right)=\left({\left|\eta \right|}^{2}-r^{2}\right)I$, we develop the following exponential map:

(91) $$\begin{array}{l} g=\exp\left(\varepsilon \beta \right)\;\mathrm{with}\;R^{2}={\left|\eta \right|}^{2}-r^{2}\\ g=\left(\begin{array}{cc} \cosh\left(\varepsilon R\right)+ir\frac{\sinh\left(\varepsilon R\right)}{R} & \eta \frac{\sinh\left(\varepsilon R\right)}{R}\\ \eta^{*}\frac{\sinh\left(\varepsilon R\right)}{R} & \cosh\left(\varepsilon R\right)-ir\frac{\sinh\left(\varepsilon R\right)}{R} \end{array}\right) \end{array}$$

We can observe that one condition is that ${\left|\eta \right|}^{2}-r^{2}>0$, then the subset to consider is given by the subset $\Lambda_{\beta }=\left\{\beta =\left(\begin{array}{cc} ir & \eta \\ \eta^{*} & -ir \end{array}\right),r\in R,\eta \in C/{\left|\eta \right|}^{2}-r^{2}>0\right\}\;$ such that $\int_{D}e^{-\langle J(z),\beta \rangle }d\lambda (z)<+\infty$. The generalized Gibbs states of the full SU(1,1) group do not exist. However, generalized Gibbs states for the one-parameter subgroups $\exp\left(\alpha \beta \right)$, $\beta \in \Lambda_{\beta }$, of the SU(1,1) group do exist. The generalized Gibbs state associated to β remains invariant under the restriction of the action to the one-parameter subgroup of $SU\left(1,1\right)$ generated by $\exp\left(\varepsilon \beta \right)$.

To go further, we will develop the Souriau Gibbs density from the Souriau moment map J(z) and the Souriau temperature $\beta \in \Lambda_{\beta }\;$. If we note $b=\frac{1}{1-{\left|z\right|}^{2}}\left[\begin{array}{c} 1\\ -z \end{array}\right]$, we can write the moment map as follows:

(92) $$J(z)==\rho \left(2Mbb^{+}-Tr\left(Mbb^{+}\right)I\right)\;\mathrm{with}\;M=\left[\begin{array}{cc} 1 & 0\\ 0 & -1 \end{array}\right]$$

We can the write the covariant Gibbs density in the unit disk given by the moment map of the Lie group $SU\left(1,1\right)$ and geometric temperature in its Lie algebra $\beta \in \Lambda_{\beta }\;$:

(93) $$p_{Gibbs}\left(z\right)=\frac{e^{-\langle J\left(z\right),\beta \rangle }}{\int_{D}e^{-\langle J\left(z\right),\beta \rangle }d\lambda (z)}\mathrm{with}\;d\lambda (z)=2i\rho \frac{dz\wedge dz^{*}}{{\left(1-{\left|z\right|}^{2}\right)}^{2}}$$

(94) $$\begin{array}{l} p_{Gibbs}\left(z\right)=\frac{e^{-\langle \rho \left(2ℑbb^{+}-Tr\left(ℑbb^{+}\right)I\right),\beta \rangle }}{\int_{D}e^{-\langle J\left(z\right),\beta \rangle }d\lambda (z)}\\ p_{Gibbs}\left(z\right)=\frac{e^{-\langle \rho \left(\begin{array}{cc} \frac{1+{\left|z\right|}^{2}}{\left(1-{\left|z\right|}^{2}\right)} & \frac{-2z^{*}}{\left(1-{\left|z\right|}^{2}\right)}\\ \frac{2z}{\left(1-{\left|z\right|}^{2}\right)} & -\frac{1+{\left|z\right|}^{2}}{\left(1-{\left|z\right|}^{2}\right)} \end{array}\right),\left(\begin{array}{cc} ir & \eta \\ \eta^{*} & -ir \end{array}\right)\rangle }}{\int_{D}e^{-\langle J\left(z\right),\beta \rangle }d\lambda (z)} \end{array}$$

To write the Gibbs density with respect to its statistical moments, we have to express the density with respect to $Q=E\left[J(z)\right]$. Then, we have to invert the relation between Q and β, to replace this last variable $\beta =\left(\begin{array}{cc} ir & \eta \\ \eta^{*} & -ir \end{array}\right)\in \Lambda_{\beta }$ by $\beta =\Theta^{-1}\left(Q\right)\in g$ where $Q=\frac{\partial \Phi \left(\beta \right)}{\partial \beta }=\Theta \left(\beta \right)\in g^{*}$ with $\Phi \left(\beta \right)=-\log\int_{D}e^{-\langle J\left(z\right),\beta \rangle }d\lambda (z)$, deduce from Legendre transform. The mean moment map is given by the following:

(95) $$\begin{array}{l} Q=E\left[J(z)\right]=E\left[\rho \left(\begin{array}{cc} \frac{1+{\left|w\right|}^{2}}{\left(1-{\left|w\right|}^{2}\right)} & \frac{-2w^{*}}{\left(1-{\left|w\right|}^{2}\right)}\\ \frac{2w}{\left(1-{\left|w\right|}^{2}\right)} & -\frac{1+{\left|w\right|}^{2}}{\left(1-{\left|w\right|}^{2}\right)} \end{array}\right)\right]\\ \mathrm{where}\;w\in D \end{array}$$

As the entropy is a Casimir function on the symplectic leaves, this also shows that these leaves are the level curves of the entropy. Transversely to the symplectic leaves, the dissipative dynamics are given by a metric bracket which remains on the leaves at constant energy. This transverse metric foliation corresponds to the level sets of energy, and the metric is given by the dual metric of the Fisher metric of information geometry, that is, the Hessian of entropy.

The structure of dissipative thermodynamics is therefore described by two transverse foliations:

- Symplectic foliation (entropy level sets) for non-dissipative dynamics, characterized by the Fisher metric and the KKS two-form on symplectic leaves.
- Metric transverse foliation (energy level sets) for dissipative dynamics, characterized by the dual Fisher metric given by the Hessian of the entropy on the metric sheets.

**Figure 18:**
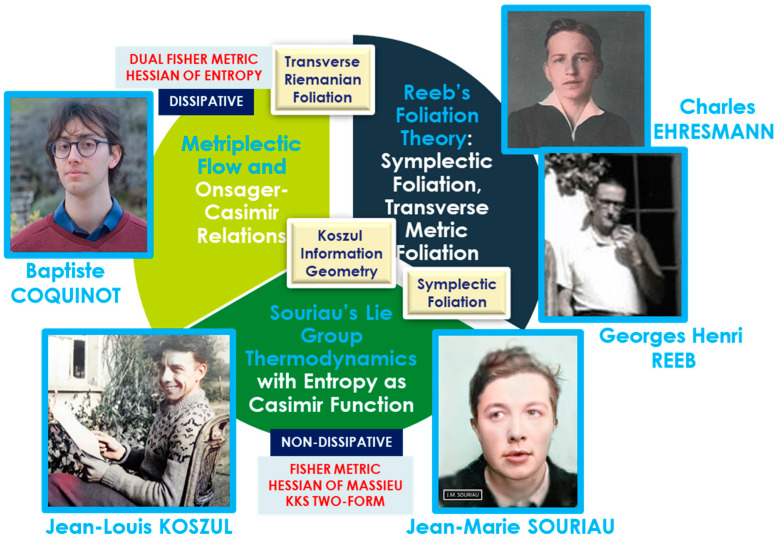
Relationship between the three models: Lie group thermodynamics by Souriau, metriplectic flow linked to the Onsager–Casimir relationships of Baptiste Coquinot, and the foliation theory of Georges Reeb and Charles Ehresmann. Source: personal picture and photos from Gallica.

In Figure 18, we provide the principal contributors to this theory illustrated at the ages of their discovery.

**Figure 19:**
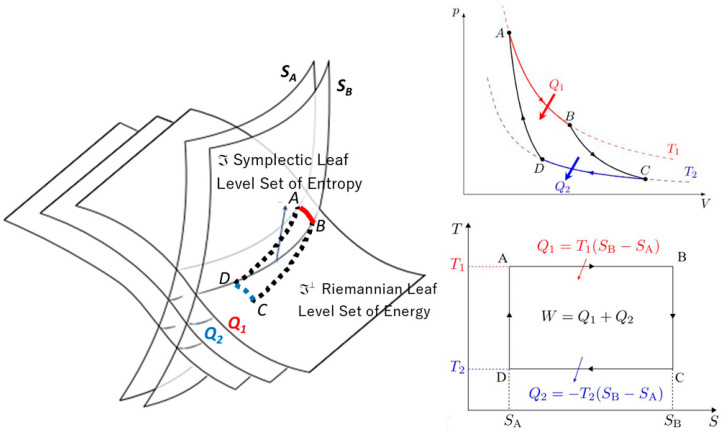
We illustrate the Carnot cycle on the Souriau’s symplectic leaves (level set of entropy) and on transverse Riemannian leaves (level set of energy): (**top-right**) Carnot cycle in temperature–pression representation, (**bottom right**) Carnot cycle in temperature–entropy representation, and (**left**) Souriau cycle on symplectic leaf (level set of entropy) and on transverse Riemannian leaf (level set of energy).

Finally, we can define Carnot’s cycle for Souriau’s Lie group thermodynamics on the symplectic leaves (levels set of entropy) and transverse Riemannian leaves (level sets of energy), as illustrated in Figure 19. The Carnot cycle is a theoretical thermodynamic cycle for a dithermic engine, made up of four reversible processes: reversible isothermal expansion, reversible adiabatic expansion (therefore isentropic), reversible isothermal compression, and reversible adiabatic compression. We can also represent Carnot cycle in a temperature–entropy diagram: AB, isothermal expansion; BC, adiabatic expansion (isentropic); CD, isothermal compression; and DA, adiabatic compression (isentropic).

## 4. Thermodynamics as a Science of Symmetry by Herbert B. Callen and Josiah Willard Gibbs

The physical phenomenon of symmetries and their physical–mathematical conceptualization traverse the history of science, as explained by Raffaele Pisano (Pisano [123]). Herbert Bernard Callen [124,125,126,127], one of the founders of the modern theory of irreversible thermodynamics, is the author of famous book *Thermodynamics and an Introduction to Thermostatistics*, published in 1960. In 1973 and 1974, he published a book chapter “A Symmetry Interpretation of Thermodynamics” and a paper on “Thermodynamics as a Science of Symmetry”, providing a new interpretation of thermodynamics from the symmetry properties of physical laws, mediated through the statistics of large systems. The fundamental laws of physics possess various symmetry properties that impose constraints and regularities on the possible properties of matter in thermodynamics. Callen made reference to Noether’s theorem:

[…] every continuous symmetry of a system implies a conservation theorem, and vice versa… The most primitive class of symmetries is the class of continuous spacetime transformations. The (presumed) invariance of physical laws under time translation implies the conservation of energy. Symmetry under spatial translation implies conservation of momentum, and rotational symmetry implies conservation of angular momentum.

(Callen [125])

He also made reference to dynamical symmetries as to gauge transformation of the electromagnetic equations (action on the scalar and vector potentials with the invariance of the electric charge). Other symmetries (baryon/lepton number, strangeness/isospin conservations in strong interactions) do not occur in conventional thermodynamic systems. The last area of symmetry is linked to the concept of broken symmetry.

About thermodynamic coordinates, without knowing Jean-Marie Souriau’s work, Herbert Bernard Callen wrote an idea related to preservation of Souriau’s moment map (geometrization of Noether theorem):

[…] The most immediately evident conserved coordinate is, of course, the energy (time-translation symmetry). Its relevance as a thermodynamic coordinate underlies the “first law” of thermodynamics. Time-translation, spatial translation, and spatial rotation symmetries are interrelated in a single class of continuous space-time symmetries. The symmetry interpretation of thermodynamics immediately suggests, then, that energy, linear momentum, and angular momentum should play fully analogous roles in thermodynamics. The equivalence of these roles is rarely evident in conventional treatments, which appear to grant the energy a misleadingly unique status. The momentum and the angular momentum are generally suppressed by restricting the theory to systems at rest, constrained by external “clamps”. Nevertheless, it is evident that in principle the linear momentum does appear in the formalism in a form fully equivalent to the energy, for relativistic considerations imply that the energy in one frame appears partially as linear momentum in another frame. Similarly, the angular momentum is only occasionally introduced explicitly into thermodynamic formalisms (as in astrophysical applications to rotating galaxies); it appears, for instance, in the “Boltzmann factor”, $\exp\left(-\beta E-\beta \Lambda .L\right)$, additively and symmetrically with the energy. To stress these facts we might well amend the first law to read that “the extended first law of thermodynamics is the symmetry of the laws of physics under space and time translations and under spatial rotation”.

(Callen [125])

**Figure 20:**
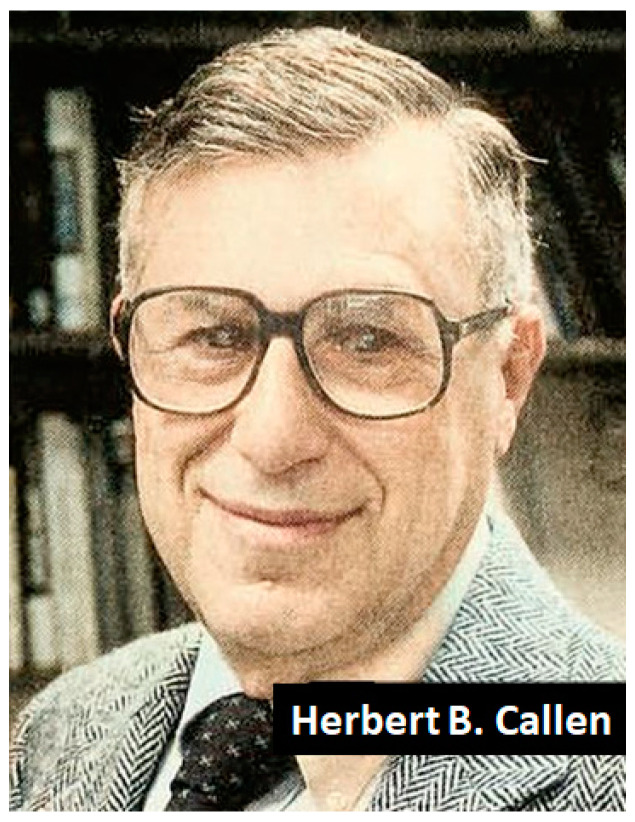
Herbert B. Callen. *Source* Wikimedia.

As Callen (portrait in Figure 20) showed that the thermodynamic coordinate symmetry has led to an extension of the first law of thermodynamics (not reduced to energy preservation but also extended to angular momentum preservation), he then considered more deeply the second and third laws of thermodynamics in relation with the emerging theory of second-order phase transitions and the Onsager extension to irreversible processes. Callen observed that equal a priori probability of states is in the form of a symmetry principle, where the entropy depends symmetrically on all permissible states.

Given unitarity symmetry of quantum mechanics among the microstates, it follows that a uniform probability density in phase space remains uniform under the intrinsic dynamics of the system. This principle determines the functional form of the entropy. The conservation of the phase space volume under an internal unitary transformation is the Boltzmann H-theorem. The interchangeability of the unitarity condition and time-reversal symmetry demonstrates that unitarity is viewed as a symmetry condition. He concluded that the central basis of the Onsager theory is the time-reversal symmetry of physical laws.

Callen emphasized the foundational importance of scale, particularly in distinguishing between thermodynamic and statistical behavior in finite versus infinite systems. He argued that the laws of thermodynamics emerge most clearly and coherently in the thermodynamic limit, wherein the number of particles and the volume tend to infinity, whilst intensive quantities such as temperature and pressure remain constant. Callen highlighted that extensive variables (such as entropy, energy, volume, etc.) must be homogeneous functions of degree one in system size. This scaling behavior underpins the Euler relation in thermodynamics and gives rise to Maxwell relations and the Legendre transform structure. In large systems, one may assume that subsystems are quasi-independent—an assumption essential for defining entropy additively, which is a core thermodynamic postulate.

In finite or small systems, however, boundary effects or interactions between subsystems become non-negligible, thereby disrupting the symmetry-based structure. Callen contrasted macroscopic determinism with the inherently probabilistic nature of small systems. Thermodynamic quantities are well defined and nearly free of fluctuations only in the limit of large systems. Finite systems, by contrast, require statistical mechanics for accurate description, as fluctuations become significant. For Callen, thermodynamics was not merely a collection of empirical laws but a deeply symmetric framework that emerges only under particular idealizations—most notably, the assumption of a large, closed system in equilibrium. In this view, system size is a prerequisite for the emergence of symmetry, rather than a mere practical consideration.

**Figure 21:**
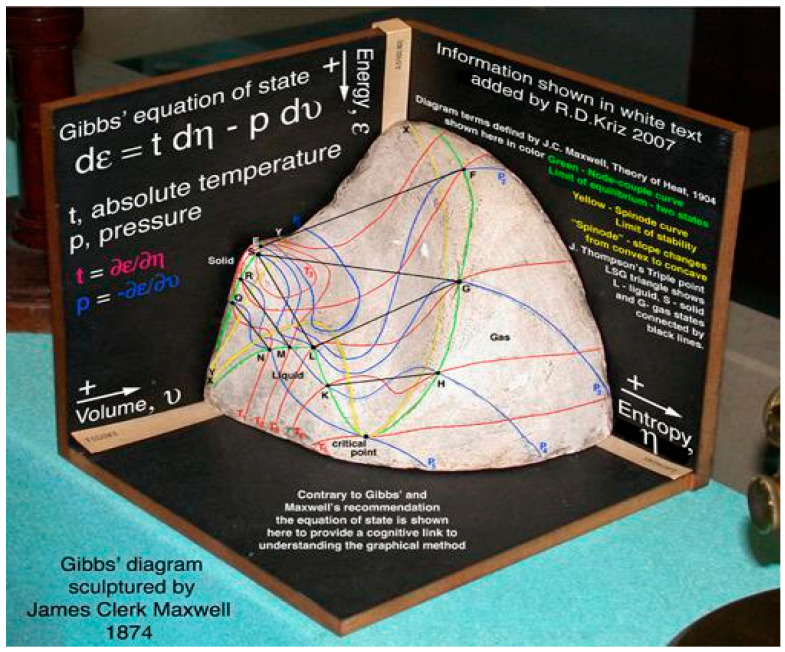
Gibbs’s geometric model of thermodynamics provided a visual and conceptual framework for understanding complex thermodynamic relationships. While modern thermodynamics primarily uses mathematical formulations, his graphical methods were pioneering in the 19th century and influenced the development of statistical mechanics and equilibrium thermodynamics.

As soon as end of 19th century, Josiah Willard Gibbs made foundational contributions to the field of thermodynamics, particularly through his employment of geometric models to represent thermodynamic systems. In his works “A Method of Geometrical Representation of the Thermodynamic Properties of Substances by Means of Surfaces” (1873) and “Graphical Methods in the Thermodynamics of Fluids”, Gibbs demonstrated how geometric surfaces could be employed to comprehend thermodynamic properties. His most significant contribution in this domain is his development of the thermodynamic surface—a graphical representation of the fundamental thermodynamic relationships. Gibbs introduced a three-dimensional geometric model to visualize thermodynamic properties in a space representing the various equilibrium states of a substance: entropy (S), volume (V), and energy (U), as illustrated in Figure 21. This surface facilitated the direct visualization of phase transitions and equilibrium conditions. His geometric model proved invaluable in explaining phase stability and the coexistence of phases via surfaces and how Maxwell’s construction could be applied to determine phase equilibria through the use of tangent planes to the thermodynamic surface. Gibbs’s graphical approach influenced subsequent developments in thermodynamics and statistical mechanics. Although graphical methods were eventually supplanted by algebraic formulations, his insights laid the foundation for the concepts of Gibbs free energy and the phase rule. His graphical methods, still widely utilized today, were devised to provide a deeper understanding of phase transitions and equilibrium states.

Josiah Willard Gibbs also formulated the theory associated with the thermodynamic state through the development of his graphical method, as expounded in his two seminal publications (Gibbs [96,97,98,99]). In the first of these works, as indicated by its title, Gibbs introduced a general graphical method that provided profound insights into the relationships between thermodynamic properties, as governed by the first and second laws of thermodynamics. In the opening paragraph of this work, Gibbs explicates as follows:

[…] Although geometrical representations of propositions in the thermodynamics of fluids are in general use, and have done good service in disseminating clear notions in this science, yet they have by no means received the extension in respect to variety and generality of which they are capable. So far as regards a general graphical method, which can exhibit at once all the thermodynamic properties of a fluid concerned in reversible processes, and serve alike for the demonstration of general theorems and the numerical solution of particular problems, it is the general if not the universal practice to use diagrams in which the rectilinear co-ordinates represent volume and pressure. The object of this article is to call attention to certain diagrams of different construction, which afford graphical methods coextensive in their applications with that in ordinary use, and preferable to it in many cases in respect of distinctness or of convenience.

(Gibbs [97])

The concept of thermodynamic potentials, as employed by Josiah Willard Gibbs, can be traced to the work of François Massieu, a distinguished French physicist. In 1869, Massieu introduced a set of potentials, including the characteristic function and entropy, which he defined as functions capable of describing the equilibrium state of a system in terms of various variables, such as pressure, temperature, and volume. Massieu’s significant insight lay in his recognition that thermodynamic quantities, such as the characteristic function and entropy, could be expressed through potentials, thereby facilitating the study and analysis of thermodynamic systems. His pioneering work laid the essential foundation for subsequent advancements in the field and notably influenced Henri Poincaré, who, upon discovering Massieu’s work, introduced the characteristic function in the context of probability theory. Poincaré’s lectures at the Sorbonne, during which he encountered Massieu’s contributions, further expanded the scope of these ideas.

Josiah Willard Gibbs subsequently developed and popularized the concept of thermodynamic potentials in the 1870s and 1880s, particularly through his seminal contributions to statistical mechanics. In his 1873 paper, “On the Equilibrium of Heterogeneous Substances”, Gibbs introduced Gibbs free energy and established the fundamental principles for the application of potentials within thermodynamics. He demonstrated how these potentials were connected to the first and second laws of thermodynamics, creating a robust mathematical framework that not only advanced the understanding of equilibrium thermodynamics but also illuminated the conditions necessary for spontaneous processes and chemical reactions. Moreover, Gibbs introduced the concept of thermodynamic equilibrium and made pivotal contributions to the phase rule and chemical potentials, enabling the study of multi-phase, multi-component systems.

It is also essential to acknowledge the contributions of Pierre Duhem, a French physicist and philosopher, whose work, while less focused on the formalism of thermodynamic potentials compared to Gibbs, provided a valuable conceptual framework for their use. Duhem advanced the idea of duality between the Massieu characteristic function and entropy, promoting the understanding that different thermodynamic potentials are merely alternative descriptions of the same thermodynamic behavior under varying constraints. His work expanded the scope of thermodynamics beyond the confines of purely mechanical systems, paving the way for its application in fields such as chemistry and engineering.

**Figure 22:**
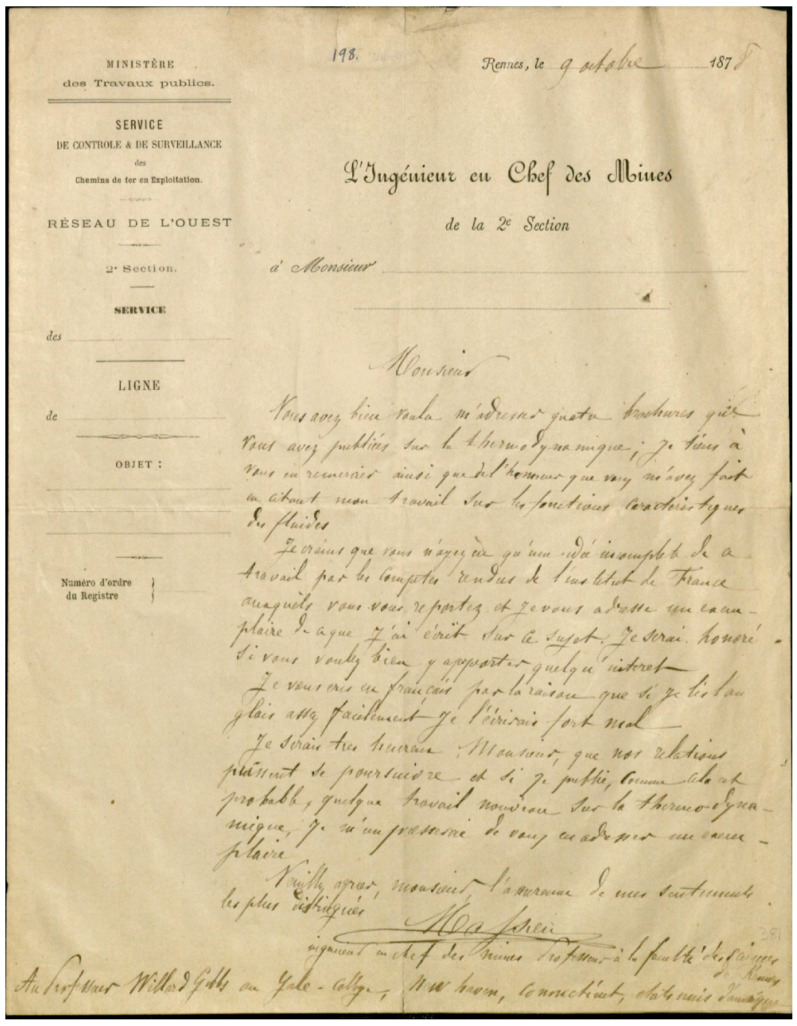
Signed letter from François Massieu (Corps des Mines) to Josiah Willard Gibbs, dated Rennes, 9 October 1878 (Gibbs Archive of Yale University).

In the following Figure 22, we provide a signed letter from François Massieu (Corps des Mines) to Josiah Willard Gibbs, dated Rennes, 9 October 1878 (Gibbs Archive of Yale University):

[…] You have been kind enough to send me four brochures that you have published on thermodynamics; I would like to thank you as well as for the honor you have done me by citing my work on the characteristic functions of fluids. I fear that you have had only an incomplete idea of this work for the reports of the Institut de France to which you refer and I am sending you a copy of what I have written on this subject. I would be honored if you would be kind enough to bring some interest to it. I am writing to you in French for the reason that if I read English quite easily, I would write it very badly. I would be very happy, Sir, if our relations could continue and if I publish, as is probable, some new work on thermodynamics, which I will hasten to send you a copy. Please accept, Sir, the assurance of my most distinguished sentiments—MASSIEU Chief Engineer of Mines, Professor at the Faculty of Sciences of Rennes to Professor Willard Gibbs, at Yale College, New Haven, Connecticut, United States of America.

(Gibbs Archive of Yale University)

As noted by Roger Balian (Balian [128]) of the French Academy of Sciences, the concept of thermodynamic potential was initially introduced in the papers of François Massieu [129,130,131,132] and subsequently expanded upon by J.W. Gibbs:

[…] Massieu’s pioneering contribution had little impact, maybe because his incentive looked technical (although he wrote in a scientific style and accounted for the most recent advances such as Clausius’s entropy). Even presently, most textbooks still ignore him, crediting Gibbs for the invention of thermodynamic potentials. In fact, as it is well known, Gibbs introduced in 1876, under the name of “fundamental function” what we now call the “free enthalpy” G(T, p, {Ni})= U − T S + pV for a fluid made of {Ni} molecules of different species. However, Gibbs himself had clearly written in a footnote: “Massieu appears to have been the first to solve the problem of representing all the properties of a body of invariable composition which are concerned in reversible processes by means of a single function”. In fact, Gibbs’s function can be regarded as an extension of Massieu’s second characteristic function to a mixture that may undergo chemical reactions. Likewise, the “free energy” F(T, p, {Ni}) = U − T S introduced in 1882 by Helmholtz appears as an extension to mixtures of Massieu’s first characteristic function. Duhem, who proposed to term all these functions “thermodynamic potentials”, properly attributes their idea to Massieu, and their introduction in thermochemistry to Gibbs. The same credits are given by Poincaré, who as Duhem presents Massieu’s functions in their modified form H and H’ of 1876. However, Planck’s potential is nothing but the original form ψ’ of Massieu’s second characteristic function; Planck, as many others, seems to have been unaware of Massieu’s work… The fact that Massieu’s original functions ψ and ψ’ of 1869 should be regarded as the most natural thermodynamic potentials, either as Legendre transforms of the entropy function or as logarithms of partition functions, is slowly getting recognition. Massieu’s name, which was not yet mentioned in Gillispie’s dictionary, now appears in Wikipedia.

(Balian [128])

**Figure 23:**
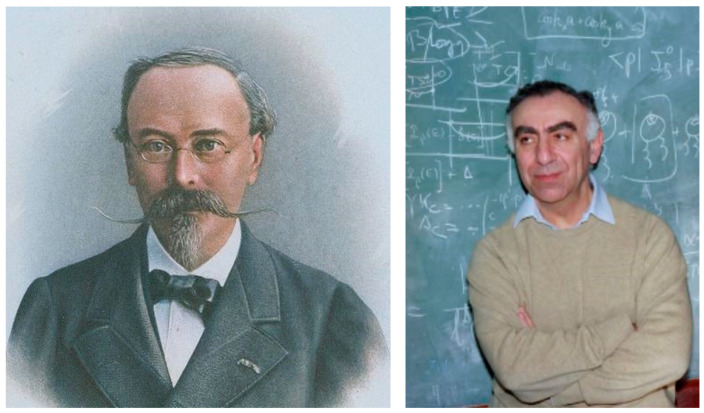
François Massieu and Roger Balian. Source Gallica and CEA IPhT.

Figure 23 features portraits of two engineers who graduated from Corps des Mines, François Massieu and Roger Balian.

We can refer to other papers by Roger Balian [133,134], L. Benayoun [135,136]. We refer to seminal works on Pfaffian form by Elie Cartan [137,138], Gaston Darboux [138,139,140] and Edouard Goursat [141]. For studies on foliations, we can also refer to Dazort [142,143,144], Ehresmann [145], Fedida [146,147,148] and Libermann [149]. For contact geometry model of thermodynamics, we give references to Jayne [150] and Mrugala [151,152]. About history of Sadi Carnot seminal works on thermodynamics, we can read Thomson [153] and Trusdell [154]. Irreversibles processes and Onsager relations are explained in [155,156,157]. For more information on François Massieu work on thermodynamics potential, good reference is Henri Poincaré Lecture at Sorbonne University [158].

## 5. Last Works of Jean-Marie Souriau on Thermodynamics

**Figure 24:**
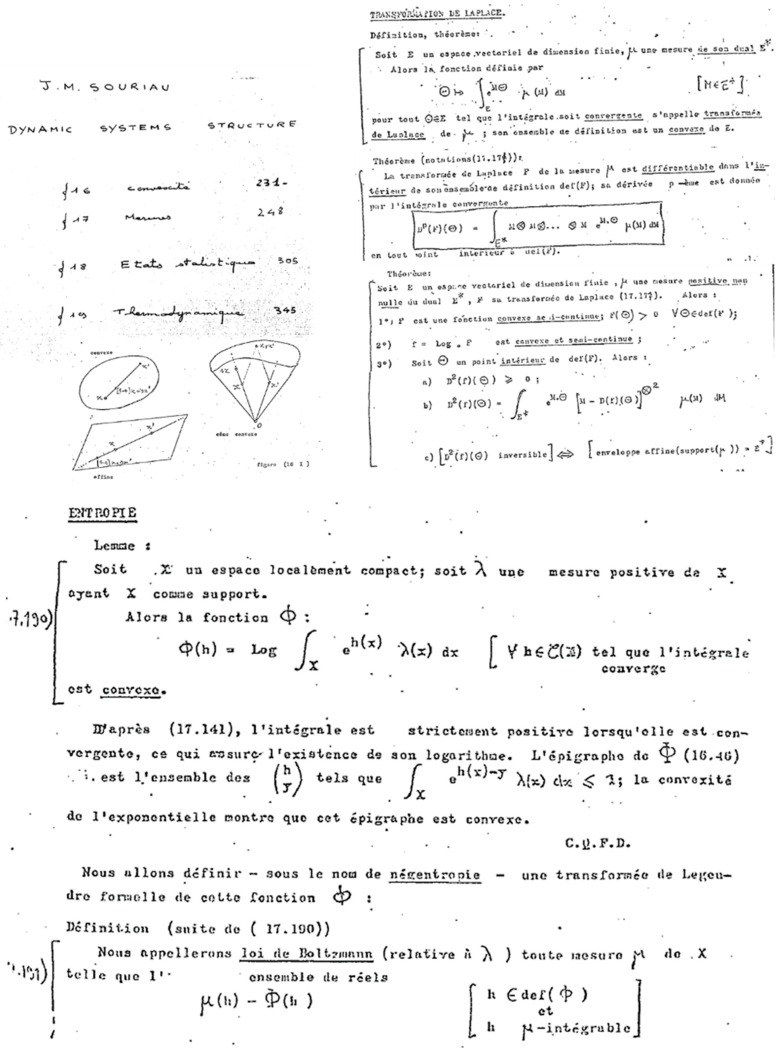
Chapter proofs for a second edition of the book *Structure of dynamic systems*.

At the time of his death, Jean-Marie had started working on a second edition of his book *Structure of Dynamic Systems*. He had planned to add four additional chapters, as illustrated in Figure 24:

[a] chapter 16 “Convexity”
[b] chapter 17 “Measurements”
[c] chapter 18 “Statistical States”
[d] chapter 19 “Thermodynamics”

Unfortunately, this work was not completed, but it would be interesting to consider a second edition of the book by appending these draft chapters which were already well advanced. There, we find a generalization of the Laplace transformation and a generalized definition of the notion of entropy.

## 6. Conclusions

We have introduced a significant link between Lie groups, thermodynamics, and information geometry by leveraging the geometric structures of Lie groups to enhance the understanding and application of thermodynamic systems within the framework of information geometry. By incorporating thermodynamic concepts in information geometry, we bridged the gap between statistical mechanics and information theory. We utilized the geometric properties of Lie groups to describe thermodynamic Gibbs states, emphasizing the definition of entropy and other thermodynamic quantities in the context of information geometry and representation theory. We extended information geometry by applying the concepts of Lie groups and thermodynamics, thus providing a deeper geometric insight into statistical manifolds. Our main contribution lies in integrating the representation theory of Lie groups and Lie algebra cohomology with the principles of thermodynamics to enrich the geometric interpretation and analysis within information geometry. This interdisciplinary approach facilitates a more profound understanding of the mathematical and physical properties of complex systems.

By mapping the states of a thermodynamic system to a symplectic manifold, we were able to describe the dynamical system using geometric structures. This foliation structure captures the dynamics of variables in thermodynamics (entropy and energy/heat), offering a more general, geometric understanding of thermodynamics. Our work builds upon statistical mechanics by relating thermodynamic quantities like entropy to geometric objects and Casimir function on symplectic foliation, generated by coadjoint orbit of the symmetry group acting on the system. This framework can be used to explain classical thermodynamic relations such as the first and second laws of thermodynamics in a more unified way.

We have applied this symplectic foliation model for dissipative thermodynamics given by metriplectic equation, used to describe systems that exhibit both reversible Hamiltonian and irreversible dissipative dynamics (such as friction or heat flow). In the metriplectic model, the main assumption was the property that entropy is a Casimir function for Poisson bracket. In the Souriau model, we have shown that, by design, the entropy is a Casimir function on symplectic leaves generated by coadjoint orbits. Our symplectic model aims to represent the dynamics of thermodynamic systems using symplectic foliation (level set of entropy) for non-dissipative dynamics and transverse Riemannian foliation (level set of energy) for dissipative dynamics. The evolution of the system through the foliated space, in a web structure, captures the balance between conservation of energy (described by the Hamiltonian part of the dynamics with Poisson bracket) and irreversibility (described by the dissipative part with metric flow bracket). The dissipative component of the metriplectic equation describes irreversible processes by entropy production, interpreting the dynamics as an evolution from one leaf to another of symplectic foliation (level set of entropy). This interprets geometrically how the system evolves toward equilibrium in a manner consistent with the second law of thermodynamics (entropy production: qualitative degradation of energy) and the first law of thermodynamics (energy preservation: qualitative preservation of energy).

This work has potential implications beyond theoretical thermodynamics. This symplectic foliation model could have impact on quantum thermodynamics: extending this classical model into the quantum domain could help bridge the gap between quantum mechanics and thermodynamic laws, particularly in systems where classical and quantum behaviors overlap, more specifically in the domain of quantum feedback for bosonic Qubit regulation, the dynamics given by Lindblad master equation, that is linear approximation of the metriplectic equation.

As statistical manifolds of information geometry are related to complete integrable systems, it would be interesting to explore a symplectic foliation model of information geometry extended for Lie groups, through Lax pairs, more specifically the natural symplectic structure on Lax pairs, expressed in terms of the algebro–geometric model. This will introduce an interplay between the analytical and the group–theoretical approaches to integrable systems.

## Figures and Tables

**Table 1:** The Lindblad and metriplectic equations share structural similarities.

Feature	Lindblad Equation	Metriplectic Equation
**Reversible Part**	$-i\left[H,\rho \right]$	$\left\{F,H\right\}$
**Dissipative Part**	$L_{k}\rho L_{k}^{+}-\frac{1}{2}\left\{L_{k}^{+}L_{k}\rho \right\}$	$\left(F,S\right)$
**Conserved Quantity**	$Tr\left(\rho \right)=1$ Energy possibly conserved	H conserved
**Entropy Production**	Entropy typically increases (for decohering system)	$\mathrm{Explicit}\;\mathrm{via}\;\left(F,S\right)$
**Underlying Structure**	Operator algebra on Hilbert space	Bracket structure (Poisson + metric)

## Abbreviations

The following abbreviations are used in this manuscript:

CITV	International Conference on Variational Theories
CNRS	Centre National de la Recherche Scientifique
ENS	Ecole Normale Supérieure
ENSET	École Nationale Supérieure de l’Enseignement Technique
ESTA	École Spéciale des Travaux Aéronautiques
GENERIC	General Equation for the Non-Equilibrium Reversible–Irreversible Coupling
KKS	Kirillov–Kostant–Souriau
ONERA	Office National d’Etudes et de Recherches Aérospatiales
SMF	Société Mathématique de France
SSD	Structure des Systèmes Dynamiques

## Symbols

The following symbols are used in this manuscript:

$Ad_{g}$	Adjoint operator
$Ad_{g}^{*}$	Coadjoint operator
$Ad_{g}^{\#}$	Affine coadjoint operator
$A\left(\tau \right)$	Rotation matrix
$\vec{b}\left(\tau \right)$	Boost vector
$C_{ij}^{k}$	Structure coefficients of the Lie algebra
$\vec{d}\left(\tau \right)$	Spatial translation vector
e	Time translation
${\vec{e}}_{z}$	Vertical vector
f	Hamiltonian function
F	Force in Newton equation
$\left\{F,G\right\}$	Poisson bracket
$\left(F,G\right)$	Metric flow bracket
$\left\{\left\{F,G\right\}\right\}$	Metriplectic bracket
G	Lie group
$g_{\beta }$	Fisher metric
g	Lie algebra
$g^{*}$	Dual of Lie algebra
H	Energy
$I\left(\beta \right)$	Fisher metric
J	Moment map
L	Lindbladian
m	Mass
r	Position
R	Rotation matrix
S	Entropy
$SU\left(1,1\right)$	Special unitary non-compact Lie group
t	Time
T	Temperature (Kelvin)
U	Moment map (in Laplace transform)
u	Potential function
$\vec{u}$	Boost vector
v	Speed
w	Element of Poincaré unit disk
$\vec{w}$	Spatial translation
$\vec{x}$	Position vector
z	Element of Poincaré unit disk
Z	Element of the Lie algebra
β	Geometric Planck temperature (element of Lie algebra)
ε	time parameter
Φ	Massieu characteristic function
$\theta \left(g\right)$	Souriau’s one-cocycle
$\left(\theta_{i},\eta_{j}\right)$	Dual natural coordinates of information geometry
λ	Liouville measure
$\tilde{\Theta }\left(X,Y\right)$	Souriau’s two-cocycle
$\sigma \left(\delta y\right)\left(\delta^{\prime }y\right)$	Lagrange–Souriau two-form
τ	Time unit
ρ	Density operator
ω	Symplectic two-form
$\psi_{\Omega }$	Laplace transform on convex cone Ω

## References

[1] Souriau J.M. (1952). Sur la Stabilité des Avions. Ph.D. Thesis.

[2] Souriau J.M., Ehresmann C., Lichnerowicz A. (1953). Géométrie symplectique différentielle. Applications. Colloque CNRS, Géométrie Différentielle, Strasbourg.

[3] Souriau J.M. (1954). Equations Canoniques et Géométrie Symplectique. Publ. Sci. Univ. D’alger A.

[4] Souria J.M. (1965). Géométrie de l’Espace des Phases. Calcul des Variations et Mécanique Quantique.

[5] Souriau J.M. (1966). Définition Covariante des Équilibres Thermodynamiques.

[6] Souriau J.M. (1967). Réalisations d’algèbres de Lie au moyen de variables dynamiques. Il Nuovo C A.

[7] Souriau J.M. (1969). Structure des Systèmes Dynamiques.

[8] Souriau J.M., Souriau J.M. (1974). Mécanique statistique, groupes de Lie et cosmologie. Colloque International du CNRS Géométrie Symplectique et Physique Mathématique.

[9] Souriau J.M. (1975). Géométrie Symplectique et Physique Mathématique.

[10] Souriau J.M., Bleuler K., Reetz A. (1977). Thermodynamique et géométrie. Differential Geometry Methods in Mathematical Physics II.

[11] Souriau J.M. (1978). Géométrie Symplectique et Physique Mathématique.

[12] Souriau J.M. (1984). Mécanique Classique et Géométrie Symplectique.

[13] Souriau J.M. (1986). La structure symplectique de la mécanique décrite par Lagrange en 1811. Math. Sci. Hum..

[14] Souriau J.M. (1990). Titres & Travaux: Principaux Thèmes de Recherche.

[15] Souriau J.M. (1995). Itinéraire d’un mathématicien—Un entretien avec Jean-Marie Souriau. Propos Recueillis par Patrick Iglesias. Le J. Maths Élèves.

[16] Souriau J.M. (1996). Grammaire de la Nature. Private Publication. http://jmsouriau.klacto.net/Souriau.2007a.pdf.

[17] Souriau J.M. (1997). Structure of dynamical systems, a symplectic view of physics. Progress in Mathematics.

[18] Souriau J.M. (2003). C’est Quantique? Donc c’est Géométrique. Série Documents de Travail (Équipe F2DS), Feuilletages-Quantification Géométrique: Textes des Journées D’étude. http://jmsouriau.klacto.net/JMS.html.

[19] Souriau J.M., Kouneiher J., Flament D., Nabonnand P., Szczeciniarz J.J. (2005). Les groupes comme universaux. Géométrie au XXe Siècle, 1930–2000. Histoire et Horizons.

[20] Souriau J.M. (2007). On Geometric Dynamics. Discret. Contin. Dyn. Syst..

[21] Wolak R.A. (1989). Foliated and associated geometric structures on foliated manifolds. Ann. Fac. Des. Sci. Toulouse 5e Sér..

[22] Morrison P.J., Updike M.H. (2023). An inclusive curvature-like framework for describing dissipation: Metriplectic 4-bracket dynamics. Phys. Rev..

[23] Carnot S. (1824). Extracts from Unpublished Notes by Sadi Carnot on Mathematics, Physics and Other Subjects.

[24] Reeb G. (1978). Structures feuilletées. Differential Topology, Foliations and Gelfand-Fuks Cohomology, Proceedings of the Symposium, Rio de Janeiro, Brazil, 5–24 January 1976.

[25] Marle C.M. (2016). From Tools in Symplectic and Poisson Geometry to, J.-M. Souriau’s Theories of Statistical Mechanics and Thermodynamics. Entropy.

[26] Marle C.M. (2018). Géométrie Symplectique et Géométrie de Poisson.

[27] Marle C.M. (2019). Projection Stéréographique et Moments, Hal-02157930, Version 1. https://hal.science/hal-02157930/document.

[28] Marle C.M. (2020). On Gibbs states of mechanical systems with symmetries. J. Geom. Symmetry Phys..

[29] Marle C.M. (2020). Examples of Gibbs States of Mechanical Systems with Symmetries. J. Geom. Symmetry Phys..

[30] Marle C.M. (2021). On Generalized Gibbs States of Mechanical Systems with Symmetries. arXiv.

[31] Marle C.M. (2021). États de Gibbs Construits au Moyen D’un Moment de L’action Hamiltonienne D’un Groupe de Lie: Signification Physique et Exemples. Diaporama Bilingue Français–Anglais, Colloque en L’honneur de Jean-Pierre Marco. https://marle.perso.math.cnrs.fr/diaporamas/GibbsStatesMomentMap.pdf.

[32] Marle C.M., Nielsen F., Barbaresco F. (2021). Gibbs States on Symplectic Manifolds with Symmetries. Geometric Science of Information. Geometric Science of Information GSI’21, Proceedings of the 5th International Conference, Paris, France, 21–23 July 2021.

[33] Gibbs J.W. (1902). Elementary Principles in Statistical Mechanics, Developed with Especial Reference to the Rational Foundation of Thermodynamics.

[34] Hadamard J. (1906). Review of Elementary Principles in Statistical Mechanics, Developed with Special Reference to the Rational Foundations of Thermodynamics by J. Willard Gibbs. Bull. Am. Math. Soc..

[35] Cartier P., Babelon O., Cartier P., Kosmann-Schwarzbach Y. (1994). Some fundamental techniques in the theory of integrable systems. Lectures on Integrable Systems.

[36] Lawson H. (1974). Foliations. Bull. Am. Math. Soc..

[37] Libermann P. (1959). Automorphismes infinitésimaux des structures symplectiques et de contact. Collection de Géométrie Différentielle Globale.

[38] Libermann P. (1983). Problèmes d’équivalence et géométrie symplectique. Astérisque.

[39] Libermann P., Dufour J.C. (1986). Sur quelques propriétés de géométrie homogène. Séminaire Sud-Rhodanien de Géométrie.

[40] Libermann P., Marle C.M. (1987). Symplectic Geometry and analytical Mechanics.

[41] Libermann P. (1989). Cartan-Darboux theorems for Pfaffian forms on foliated manifolds. Proceedings of the VIth International Colloquium Differential Geometry, 1989.

[42] Libermann P. (1991). Legendre Foliations on Contact Manifolds. Differ. Geom. Its Appl..

[43] Libermann P., Kouneiher J., Flament D., Nabonnand P., Szczeciniarz J.J. (2005). La géométrie différentielle d’Elie Cartan à Charles Ehresmann et André Lichnerowicz. Géométrie au XXe Siècle, 1930–2000. Histoire et Horizons.

[44] Pang M.Y. (1990). The Structure of Legendre Foliations. Trans. Am. Math. Soc..

[45] Lichnerowicz A. (1983). Formes caractéristiques d’un feuilletage et classes de cohomologie de l’algèbre des vecteurs tangents à valeurs dans les formes normales. C. R. Acad. Sci. Paris.

[46] Vojta G. (1990). Symplectic Formalism for the Thermodynamics of Irreversible Processes. Ann. Der Phys..

[47] Arnold V. (1966). Sur la géométrie différentielle des groupes de Lie de dimension infinie et ses applications à l’hydrodynamique des fluides parfaits. Ann. L’inst. Fourier.

[48] Arnold V.I., Givental A.B., Arnold V.I., Novikov S.P. (1990). Symplectic Geometry. Dynamical Systems IV Symplectic Geometry and Its Applications.

[49] Khesin B.A., Tabachnikov S.L. (2014). Arnold Swimming Against the Tide.

[50] Lichnerowicz A., Rund H., Forbes W. (1976). Variétés symplectiques, canoniques et systèmes dynamiques. Topics in Differential Geometry, Volume in Honour of ET Davies.

[51] Lichnerowicz A. (1982). Géométrie différentielle des variétés de contact. J. Math. Pures Appl..

[52] Lichnerowicz A. (1982). Variétés de Poisson et feuilletages. Ann. Fac. Sci. Toulouse.

[53] Lichnerowicz A., Barut A.O. (1983). Quantum Mechanics and déformations of Geometrical Dynamics. Quantum Theory, Groups, Fields and Particles.

[54] Lichnerowicz A., Van-Tan T. (1983). Feuilletages, géométrie riemannienne et géométrie symplectique. Ibidem.

[55] Lichnerowicz A. (1983). Physique mathématique, Cours du Collège de France 1982–1983. Inst. De France.

[56] Kirillov A.A. (1974). Eléments de la Théorie des Représentations.

[57] Kirillov A.A. (2004). Lectures on the orbit method. Graduate Studies in Mathematics.

[58] Kapranov M. (2011). Thermodynamics and the moment map. arXiv.

[59] Nencka H., Streater R.F. (1999). Information Geometry for some Lie algebras. Infin. Dimens. Anal. Quantum Probab. Relat. Top..

[60] Pavlov V.P., Sergeev V.M. (2008). Thermodynamics from the Differential Geometry Standpoint. Theor. Math. Phys..

[61] Casimir H.B.G. (1945). On onsager’s principle of microscopic reversibility. Rev. Mod. Phys..

[62] Bachelard G. (1973). La propagation thermique dans les solides. Étude Sur L’évolution D’un Problème de Physique.

[63] Cosserat O. (2023). Theory and Construction of Structure Preserving Integrators in Poisson Geometry. Differential Geometry.

[64] Hubmer G.F., Titulaer U.M. (1987). The Onsager-Casimir relations revisited. J. Stat. Phys..

[65] Coquinot B., Morrison P.J. (2020). A General Metriplectic Framework with Application to Dissipative Extended Magnetohydrodynamics. J. Plasma Phys..

[66] Vallée C., de Saxcé G., Marle C.M. (2012). Hommage à Jean-Marie Souriau. Gaz. Des Math..

[67] Françoise J.P. Systèmes Dynamiques appliqués aux Oscillations. Proceedings of the 21ème Congrès Français de Mécanique.

[68] Noether E. (1918). Invariante variationsprobleme. Nachrichten Ges. Der Wiss. Zu Gott. Abh. Math.-Phys. Klasse.

[69] Audin M. (2008). Differential Geometry, Strasbourg, 1953. Not. AMS.

[70] de Saxcé G., Vallée C. (2012). Bargmann group, momentum tensor and Galilean invariance of Clausius–Duhem inequality. Int. J. Eng. Sci..

[71] de Saxcé G., Vallée C. (2016). Galilean Mechanics and Thermodynamics of Continua.

[72] de Saxcé G. (2016). Link between Lie Group Statistical Mechanics and Thermodynamics of Continua. Entropy.

[73] de Saxcé G., Nielsen F., Barbaresco F. (2019). Euler-Poincaré equation for Lie groups with non null symplectic cohomology. Application to the mechanics. Geometric Science of Information GSI’19, Proceedings of the 4th International Conference, Toulouse, France, 27–29 August 2019.

[74] de Saxcé G., Marle C.M., Barbaresco F., Nielsen F. (2022). Structure des Systèmes Dynamiques Jean-Marie Souriau’s Book 50th Birthday. Geometric Structures of Statistical Physics, Information Geometry, and Learning.

[75] Lagrange J.L. (1855). Mécanique Analytique.

[76] Bourguignon J.P. Jean-Marie Souriau and Symplectic Geometry, Souriau’19 conference, 50th Birthday of Jean-Marie Souriau’s Book, Paris, 2019. https://www.youtube.com/watch?v=93hFolIBo0Q&t=3s.

[77] Gallisot F. (1952). Les formes extérieures en mécanique. Ann. L’inst. Fourier.

[78] Coleman C.P. (1994). The Search for Stable Equilibria on Coadjoint Orbits and Applications to Dissipative Processes.

[79] Barbaresco F. (2018). Higher Order Geometric Theory of Information and Heat Based on Poly-Symplectic Geometry of Souriau Lie Groups Thermodynamics and Their Contextures: The Bedrock for Lie Group Machine Learning. Entropy.

[80] Barbaresco F., Nielsen F. (2019). Jean–Louis Koszul and the elementary structures of information geometry. Geometric Structures of Information.

[81] Barbaresco F., Lachieze-Rey M. (2019). Lie Groups Thermodynamics & Souriau-Fisher Metric. Proceedings of the Souriau 2019 Conference.

[82] Barbaresco F., Nielsen F., Barbaresco F. (2019). Souriau Exponential Map Algorithm for Machine Learning on Matrix Lie Groups. Geometric Science of Information GSI’19, Proceedings of the 4th International Conference, GSI 2019, Toulouse, France, 27–29 August 2019.

[83] Barbaresco F. (2020). Lie Group Statistics and Lie Group Machine Learning Based on Souriau Lie Groups Thermodynamics & Koszul-Souriau-Fisher Metric: New Entropy Definition as Generalized Casimir Invariant Function in Coadjoint Representation. Entropy.

[84] Barbaresco F., Nielsen F., Barbaresco F. (2021). Souriau-Casimir Lie Groups Thermodynamics and Machine Learning. Geometric Structures of Statistical Physics, Information Geometry, and Learning.

[85] Barbaresco F. (2021). Koszul lecture related to geometric and analytic mechanics, Souriau’s Lie group thermodynamics and information geometry. Inf. Geom. J..

[86] Barbaresco F., Nielsen F. (2021). Invariant Koszul Form of Homogeneous Bounded Domains and Information Geometry Structures. Progress in Information Geometry.

[87] Barbaresco F., Nielsen F., Barbaresco F. (2021). Jean-Marie Souriau’s Symplectic Model of Statistical Physics: Seminal Papers on Lie Groups Thermodynamics—Quod Erat Demonstrandum. Geometric Structures of Statistical Physics, Information Geometry, and Learning.

[88] Barbaresco F., Nielsen F., Barbaresco F. (2021). Archetypal Model of Entropy by Poisson Cohomology as Invariant Casimir Function in Coadjoint Representation and Geometric Fourier Heat Equation. Geometric Science of Information, Proceedings of the 5th International Conference, GSI 2021, Paris, France, 21–23 July 2021.

[89] Barbaresco F. (2022). Symplectic theory of heat and information geometry. Handbook of Statistics.

[90] Barbaresco F. (2022). Symplectic Foliation Structures of Non-Equilibrium Thermodynamics as Dissipation Model: Application to Metriplectic Nonlinear Lindblad Quantum Master Equation. Entropy.

[91] Barbaresco F. Densité de probabilité gaussienne à maximum d’Entropie pour les groupes de Lie basée sur le modèle symplectique de Jean-Marie Souriau. Proceedings of the GRETSI’22 Conference.

[92] Barbaresco F. Théorie symplectique de l’Information et de la chaleur: Thermodynamique des groupes de Lie et définition de l’Entropie comme fonction de Casimir. Proceedings of the GRETSI’22 Conference.

[93] Barbaresco F., Freeden W., Zuhair Nashed M. (2022). Entropy Geometric Structure as Casimir Invariant Function in Coadjoint Representation: Geometric Theory of Heat & Information Geometry Based on Souriau Lie Groups Thermodynamics and Lie Algebra Cohomology. Frontiers in Entropy Across the Disciplines.

[94] Barbaresco F., Freeden W., Zuhair Nashed M. (2022). Souriau Entropy Based on Symplectic Model of Statistical Physics: Three Jean-Marie Souriau’s Seminal Papers on Lie Groups Thermodynamics. Frontiers in Entropy Across the Disciplines.

[95] Barbaresco F., Nielsen F., Barbaresco F. (2023). Symplectic Foliation Transverse Structure and Libermann Foliation of Heat Theory and Information Geometry. Geometric Science of Information GSI’23, Proceedings of the 6th International Conference, St. Malo, France, 30 August–1 September 2023.

[96] Gibbs J.W. (1873). Graphical Methods in the Thermodynamics of Fluids. Trans. Conn. Acad..

[97] Gibbs J.W. (1873). A Method of Geometrical Representation of the Thermodynamic Properties of Substances by Means of Surfaces. Transactions of the Connecticut Academy, Vol. II, Part 2.

[98] Gibbs J.W. (1875). Equilibrium of Heterogeneous Substances. Trans. Conn. Acad..

[99] Gibbs J.W. (1906). On the Equilibrium of Heterogeneous Substances.

[100] Kozlov V.V. (2004). Gibbs and Poincaré Statistical Equilibria in Systems with Slowly Varying Parameters. Dokl. Math..

[101] Balian R. (2007). From Microphysics to Macrophysics (Methods and Applications of Statistical Physics), Vol I.

[102] Martinet J., Reeb G., Maslov V. (1973). Sur une généralisation des structures feuilletées de codimension 1. Dynamical Systems.

[103] Reeb G. (1952). Sur certaines propriétés topologiques des trajectoires des systèmes dynamiques. Acad. R. Belg..

[104] Reeb G. (1956). Sur la théorie générale des systèmes dynamiques. Ann. L’inst. Fourier.

[105] Reeb G. (1959). Structures feuilletées. Bull. Soc. Math. Fr..

[106] Reeb G., Ehresmann C., Thom R., Libermann P. (1964). Structures feuilletées. Colloques Internationaux Du Cnrs (CIDC).

[107] Haefliger A., Kouneiher J., Flament D., Nabonnand P., Szczeciniarz J.J. (2016). Naissance des feuilletages, d’Ehresmann-Reeb à Novikov. Géométrie au XXe Siècle, 1930–2000. Histoire et Horizons.

[108] Molino P. (1989). Dualité symplectique, feuilletage et géométrie du moment. Publ. Mat..

[109] Molitor M. (2021). Kähler toric manifolds from dually flat spaces. arXiv.

[110] Condevaux M., Dazord P., Molino P. (1988). Géométrie du moment. Publications du Département de Mathématiques de Lyon, Fascicule 1B, Séminaire Sud-Rhodanien, 1988.

[111] Reinhart B.L. (1983). Differential Geometry of Foliations.

[112] Basart H., Lichnerowicz A. (1982). Variétés de poisson et star-produits tangentiels. Ibidem 1.

[113] Hamoui A., Lichnerowicz A. (1982). Sur la quantification d’un système dynamique à hamiltonien dépendant du temps. C. R. Acad. Sci. Paris.

[114] Lie S. (1876). Allgemeine Theorie der partiellen Differentialgleichungen erster Ordnung. Math. Ann..

[115] Lie S. (1877). Allgemeine Theorie der partiellen Differentialgleichungen erster Ordnung (Zweite Abhandlung). Math. Ann..

[116] Lie S. (1893). Transformationsgruppen, I, II, III.

[117] Albert C. (1989). Le théorème de réduction de Marsden-Weinstein en géométrie cosymplectique et de contact. J. Geom. Phys..

[118] Jaynes E.T. (1957). Information Theory and Statistical Mechanics. Phys. Rev..

[119] Jaynes E.T. (1957). Information Theory and Statistical Mechanics II. Phys. Rev..

[120] Nehorosev N.N. (1972). Action-angle variables and their generalizations. Transl. Mosc. Math. Soc..

[121] Delzant T. (1986). Variables Actions-Angles non Commutatives et Exemples D’images Convexes de L’application Moment.

[122] Delzant T. (1986). Hamiltoniens périodiques et images convexes de l’application moment. Bull. Soc. Math. Fr..

[123] Pisano R. (2024). Brief summaries on symmetries in the history of physics–mathematics: James Clerk Maxwell (1865–1873), Emmy Noether (1915–1918) and Albert Einstein (1905–1926). J. Phys. Conf. Ser. IOP Oxf..

[124] Callen H.B., Domingos J.D.D. (1973). A Symmetry Interpretation of Thermodynamics. Foundations of Continuum Thermodynamics.

[125] Callen H.B. (1974). Thermodynamics as a Science of Symmetry. Found. Phys..

[126] Callen H.B. (1985). Thermodynamics and An Introduction to Thermostatistics.

[127] Carnot S. (1824). Réflexions sur la Puissance Motrice du Feu et sur les Machines Propres à Développer Cette Puissance.

[128] Balian R. (2015). François Massieu et les potentiels thermodynamiques. Évolution des Disciplines et Histoire des Découvertes.

[129] Massieu F. (1869). Sur les Fonctions caractéristiques des divers fluides. C. R. L’acad. Sci. Paris.

[130] Massieu F. (1869). Addition au précédent Mémoire sur les Fonctions caractéristiques. C. R. L’acad. Sci. Paris.

[131] Massieu F. (1873). Exposé des Principes Fondamentaux de la Théorie Mécanique de la Chaleur (Note Destinée à Servir D’introduction au Mémoire de L’auteur sur les Fonctions Caractéristiques des Divers Fluides et la Théorie des Vapeurs).

[132] Massieu F. (1876). Thermodynamique: Mémoire sur les Fonctions Caractéristiques des Divers Fluides et sur la Théorie des Vapeurs.

[133] Balian R., Alhassid Y., Reinhardt H. (1986). Dissipation in many-body systems: A geometric approach based on information theory. Phys. Rep..

[134] Balian R., Valentin P. (2001). Hamiltonian structure of thermodynamics with gauge. Eur. Phys. J. B Condens. Matter Complex. Syst..

[135] Benayoun L. (1999). Méthodes Géométriques Pour L’étude des Systèmes Thermodynamiques et la Génération D’équations D’état. Ph.D. Thesis.

[136] Benayoun L., Valentin P. (2022). Evolution of equations of state through contact transformations. Fluid. Phase Equilib..

[137] Cartan E. (1899). Sur certaines expressions différentielles et le problème de Pfaff. Ann. Sci. L’école Norm. Supérieure.

[138] Cartan E. (1958). Leçons sur les Invariants Intégraux.

[139] Darboux G. (1882). Sur le problème de Pfaff (Partie 1). Bull. Sci. Math. Astron..

[140] Darboux G. (1882). Sur le problème de Pfaff (Partie 2). Bull. Sci. Math. Astron..

[141] Goursat E. (1922). Leçons sur les Problèmes de Pfaff.

[142] Dazord P. (1983). Feuilletages et mécanique hamiltonienne. Publ. Du Dép. Math. (Lyon).

[143] Dazord P., Molino P. (1988). Gamma-Structures poissonniennes et feuilletages de Libermann. Publ. Du Dép. Math. (Lyon).

[144] Dazord P., Delzant T. (1987). Le problème général des variables actions-angles. J. Differ. Geom..

[145] Ehresmann C. (1951). Sur la théorie des variétés feuilletées. Rend. Di Mat..

[146] Fedida E. (1973). Feuilletages du Plan, Feuilletages de Lie. Ph.D. Thesis.

[147] Fedida E. (1974). Sur l’existence des feuilletages de Lie. C.-R. L’acad. Sci. Paris.

[148] Fedida E., Schweitzer P.A. (1978). Sur la théorie des feuilletages associée au repère mobile: Cas des feuilletages de lie. Differential Topology, Foliations and Gelfand-Fuks Cohomology.

[149] Libermann P. (1954). Sur Ie problème d’équivalence de certaines structures infinitésimales regulières. Ann. Mat. Pura Ed Appl..

[150] Jayne N. (1992). Legendre Foliations on Contact Metric Manifolds. Ph.D. Thesis.

[151] Mrugala R. (1978). Geometrical formulation of equilibrium phenomenological thermodynamics. Rep. Math. Phys..

[152] Mrugala R. (2000). On contact and metric structures on thermodynamic spaces. Math. Asp. Quantum Inf. Quantum Chaos.

[153] Thomson W. (1849). XXXVI.—An Account of Carnot’s Theory of the Motive Power of Heat; with Numerical Results deduced from Regnault’s Experiments on Steam. Trans. R. Soc. Edinb..

[154] Trusdell C. (1980). The Tragicomical History of Thermodynamics 1822–1854.

[155] Onsager L. (1931). Reciprocal relations in irreversible processes I. Phys. Rev..

[156] Onsager L., Machlup S. (1953). Fluctuations and Irreversible Processes. Phys. Rev..

[157] Onsager L., Machlup S. (1953). Fluctuations and Irreversible Processes II. Systems with Kinetic Energy. Phys. Rev..

[158] Poincaré H. (1908). Thermodynamique; Cours de Sorbonne 2nd Édition Revue et Corrigée.

